# Frequency-Dependent Action of Neuromodulation

**DOI:** 10.1523/ENEURO.0338-21.2021

**Published:** 2021-11-08

**Authors:** Anna C. Schneider, David Fox, Omar Itani, Jorge Golowasch, Dirk Bucher, Farzan Nadim

**Affiliations:** Federated Department of Biological Sciences, New Jersey Institute of Technology and Rutgers University, Newark, NJ 07102

**Keywords:** calcium, central pattern generator, modeling, neuromodulation, stomatogastric

## Abstract

In oscillatory circuits, some actions of neuromodulators depend on the oscillation frequency. However, the mechanisms are poorly understood. We explored this problem by characterizing neuromodulation of the lateral pyloric (LP) neuron of the crab stomatogastric ganglion (STG). Many peptide modulators, including proctolin, activate the same ionic current (*I*_MI_) in STG neurons. Because *I*_MI_ is fast and non-inactivating, its peak level does not depend on the temporal properties of neuronal activity. We found, however, that the amplitude and peak time of the proctolin-activated current in LP is frequency dependent. Because frequency affects the rate of voltage change, we measured these currents with voltage ramps of different slopes and found that proctolin activated two kinetically distinct ionic currents: the known *I*_MI_, whose amplitude is independent of ramp slope or direction, and an inactivating current (*I*_MI-T_), which was only activated by positive ramps and whose amplitude increased with increasing ramp slope. Using a conductance-based model we found that *I*_MI_ and *I*_MI-T_ make distinct contributions to the bursting activity, with *I*_MI_ increasing the excitability, and *I*_MI-T_ regulating the burst onset by modifying the postinhibitory rebound in a frequency-dependent manner. The voltage dependence and partial calcium permeability of *I*_MI-T_ is similar to other characterized neuromodulator-activated currents in this system, suggesting that these are isoforms of the same channel. Our computational model suggests that calcium permeability may allow this current to also activate the large calcium-dependent potassium current in LP, providing an additional mechanism to regulate burst termination. These results demonstrate a mechanism for frequency-dependent actions of neuromodulators.

## Visual Abstract

**Figure:**
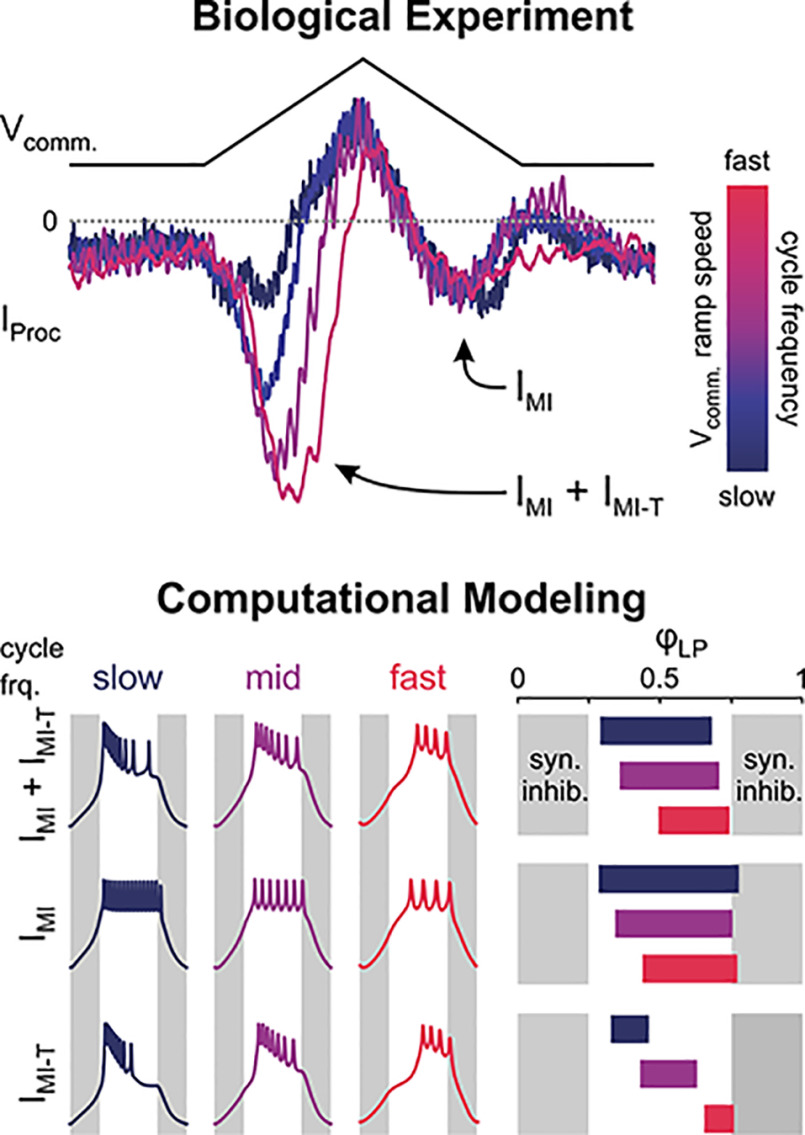

## Significance Statement

Oscillatory neurons respond to synaptic input in complex ways that depend on the polarity, amplitude, and rate of the input, and intrinsic properties of the cell. As a result, neuromodulator inputs that activate voltage-gated ionic currents can have indirect and state-dependent effects. We show that when a target of neuromodulation is a transient ionic current, an additional layer of complexity of the response emerges in which the oscillation frequency and the indirect influence of other ionic currents shape the amplitude and temporal properties of the neuronal response to the modulator.

## Introduction

Neuron and network activity is tuned by neuromodulators that influence neuronal excitability and synaptic function, often through G-protein-coupled receptor signaling (Marder, 2012; Nadim and Bucher, 2014; Burke and Bender, 2019). An important aspect of this is the modulation of the gating properties of voltage-gated ion channels. The effect of activation or modulation of a voltage-gated current on the activity and response properties of a neuron depends on the complement, magnitude, and temporal trajectory of other currents, as neuron output is shaped by complex nonlinear interactions of multiple ionic mechanisms and their dependence and effect on the membrane potential (Taylor et al., 2009). As such, the contribution of each voltage-gated ionic current critically depends on the voltage trajectory, i.e., both the range of the membrane potential and its time-dependent changes, which are in turn influenced by synaptic inputs.

This dependence is particularly apparent in rhythmically active neurons. For example, thalamocortical neurons produce bursting oscillations arising from the T-type calcium current (*I*_CaT_), but only when the baseline membrane potential is hyperpolarized (Steriade and Contreras, 1995; Amarillo et al., 2014). Additionally, responses of bursting neurons critically depend on strength, frequency, short-term plasticity, and temporal trajectory of their synaptic input (Martinez et al., 2019). In many circuits, neurons burst on rebound from synaptic inhibition (Huguenard and McCormick, 2007; Bucher et al., 2015). The shape and strength of inhibitory input to such a neuron would produce very different effects if the rebound were because of a persistent current such as the persistent sodium current (*I*_NaP_), a transient current such as *I*_CaT_, or a hyperpolarization-activated current such as *I*_h_. While persistent currents show little dependence on the history of activity, the voltage and time dependence of channel inactivation makes the contribution of transient currents dependent on oscillation frequency and prior activation (Baukrowitz and Yellen, 1995; Roeper et al., 1997; Armstrong and Roberts, 2001; Carter and Bean, 2011).

Interactions between ionic currents and the amplifying or inhibitory effects of neuromodulators on individual currents can generate great flexibility in circuit operation. However, because of the complexity of such interactions, neuromodulation of any individual ionic current may produce effects that are not easily predictable. Here, we explored how the actions of neuromodulators may depend on the membrane potential trajectory of the target neuron and on the frequency of inputs it receives in an oscillatory network.

**Figure 1.**
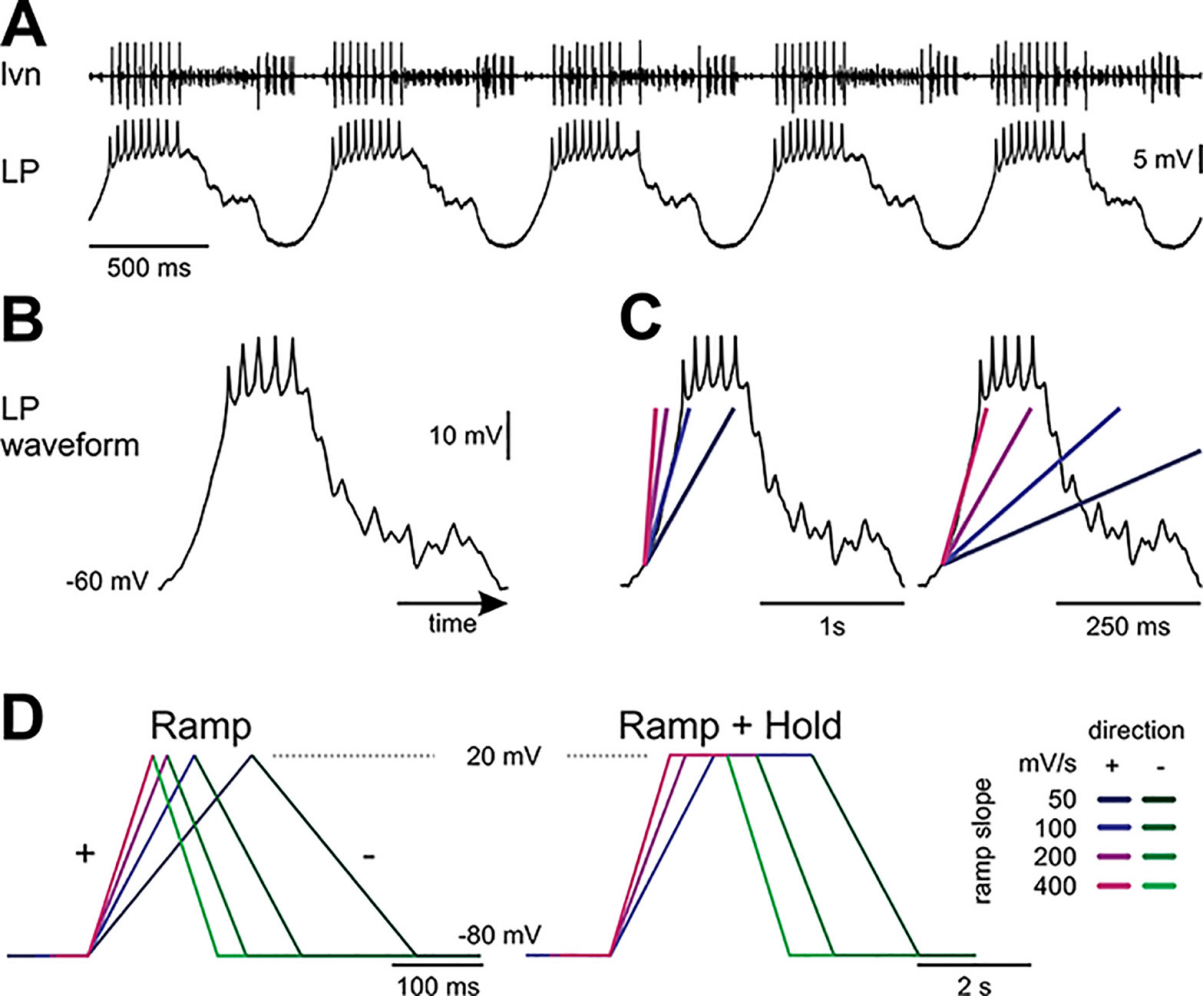
Voltage-clamp paradigms. ***A***, Extracellular lateral ventricular nerve (*lvn*) and intracellular LP neuron recording. The *lvn* carries the axons of several neurons participating in the triphasic pyloric rhythm. The largest action potentials in the lvn recording are from the LP neuron. Different units can be recognized by different amplitudes. The follower neuron LP oscillates in time with the pyloric rhythm because of strong periodic inhibitory input and fires bursts of action potentials on rebound from inhibition. ***B***, Canonical waveform of LP. This prerecorded waveform was used to drive LP’s membrane potential in voltage clamp to mimic a realistic LP neuron activity. In the voltage-clamp experiments, the slow-wave oscillation was scaled so that it ranged from a trough potential of −60 mV to −20 mV. ***C***, LP’s depolarization rate can be approximated with different slopes (colored lines) for different pyloric cycle periods. ***D***, These slopes were used to construct symmetrical ramp or ramp-and-hold stimuli to sample *I-V* relationships for proctolin-activated currents at different polarization rates, which roughly correspond to different cycle periods (Extended Data Fig. 1-1). The same color-code for slopes is used for all figures with purple colors for positive (+) ramps and green colors for negative (–) ramps.

Neuromodulation has been extensively studied in oscillatory circuits such as the central pattern generators (CPGs) of the brain stem respiratory system (Doi and Ramirez, 2008; Ramirez and Baertsch, 2018), and the crustacean stomatogastric nervous system (STNS; Marder and Bucher, 2007; Stein, 2009; Daur et al., 2016), where a multitude of neuromodulators influence circuit activity patterns. The crustacean pyloric circuit is a well-studied CPG that produces oscillations in a broad range of frequencies. Pyloric oscillations are driven by pacemaker neurons that drive all follower neurons with strong inhibitory synapses. The follower neurons rebound from this inhibition to produce a burst of action potentials (Fig. 1*A*). Excitatory neuropeptides and muscarinic agonists activate a fast, voltage-dependent persistent current (the modulator-activated inward current, *I*_MI_), which is crucial for oscillatory activity in pacemaker neurons (Golowasch and Marder, 1992; Goaillard et al., 2010; Zhao et al., 2010; Bose et al., 2014). However, the effect of *I*_MI_ on follower neurons, which can provide feedback to pacemaker neurons, is less understood. The strength, shape and frequency of inhibition influence the voltage trajectory of the burst response of follower neurons (Harris-Warrick et al., 1995a,b; Hooper, 1998; Kloppenburg et al., 1999; Martinez et al., 2019). Because this voltage trajectory determines the activation of all voltage-gated ionic currents, it also controls the levels of currents regulated by such modulators. Meanwhile, the modulatory current in turn influences the voltage trajectory.

10.1523/ENEURO.0338-21.2021.f1-1 Extended Data Figure 1-1 Depolarization rates for different cycle periods of the unitary realistic LP voltage waveform. Download Figure 1-1, XLSX file.

Among the best studied neuropeptide modulators of the pyloric circuit is proctolin, which activates *I*_MI_ in all but one pyloric neuron type (Swensen and Marder, 2001), including the lateral pyloric (LP) neuron. To address whether the actions of proctolin on the follower LP neuron vary dependent on circuit frequency, we used a variety of voltage-clamp paradigms. We measured the current–voltage (*I-V*) relationship of proctolin-activated currents with voltage ramps of different slopes that correspond to LP depolarization rates at different cycle frequencies and used realistic LP neuron waveforms applied at different cycle frequencies. We found that the proctolin-activated current is more complex than previously determined and contains an inactivating component. Using a computational model, we explored the possible role of this inactivating component and show that its frequency dependence can produce state-dependent effects at the circuit level.

## Materials and Methods

### Solutions

*Cancer borealis* saline contained the following: 440 mm NaCl, 26 mm MgCl_2_, 13 mm CaCl_2_, 11 mm KCl, 10 mm Tris base, and 5 mm maleic acid, buffered to pH 7.4–7.5. Custom synthesized Proctolin (RS Synthesis, sequence RYLPT) was dissolved in distilled water and stored as 10^−3^ m aliquots at −20°C. Immediately before usage, proctolin stock was diluted in saline to a final concentration of 10^−6^ m. To prevent neurons from spiking during voltage-clamp experiments, we added 10^−7^ m tetrodotoxin (TTX; Alomone Labs), stored as 10^−4^ m stock solution in distilled water at 4°C, to the saline to block voltage-dependent Na^+^ currents. In some experiments, we added 2 × 10^−4^ m Cd^2+^ to the saline to block Ca^2+^ channels (Golowasch and Marder, 1992).

### Electrophysiology

Male Jonah crabs (*C. borealis*) were anesthetized by placing them in ice for at least 30 min. Their STNS was dissected as described previously (Gutierrez and Grashow, 2009) and pinned dorsal side up in a Sylgard (Ellsworth Adhesives) lined Petri dish. The sheath around the stomatogastric ganglion (STG) was removed with fine tungsten pins to facilitate electrode penetration and chemical uptake by the neurons. The STG was constantly perfused with 10–13°C saline during the experiments. We recorded the pyloric rhythm for cell identification with stainless steel pin electrodes inside Vaseline wells built around the lateral ventricular nerve (*lvn*). Extracellular electrodes were connected to a differential AC amplifier (Model 1700, AM Systems). LP was identified by matching its intracellularly recorded activity to the extracellularly recorded pyloric rhythm (Fig. 1*A*).

We used the two-electrode voltage-clamp technique to measure proctolin-activated currents in the soma of the LP neuron. Electrodes were pulled from borosilicate capillaries with filament and filled with 0.6 m K_2_SO_4_ + 20 mm KCl (resistance: 20–25 MΩ). Intracellular signals were amplified (Axoclamp 900A, Molecular Devices), and all recordings digitized at 5 kHz (Digidata 1440A, Molecular Devices) and recorded with Clampex 10.6 (Molecular Devices). Voltage-clamp waveforms were created with MATLAB (R2019a; MathWorks) and delivered via Clampex.

To measure the proctolin-activated currents, we removed all intrinsic neuromodulators by transecting the stomatogastric nerve (nerve that carries the axons of all modulatory projection neurons from central ganglia) and/or by perfusing the STG with 10^−7^ m TTX saline to block action potentials, which prevents transmitter release from the terminals of modulatory projection neurons.

To apply proctolin, we built a separate Vaseline well around the STG and superfused the drug only on the STG to reduce application and wash times. We waited at least 10 min after cessation of action potentials before starting voltage-clamp measurements in control condition. Proctolin-activated currents (henceforth proctolin currents, *I*_Proc_) were calculated as the difference between total currents measured in normal saline and in the presence of 10^−6^ m proctolin (*I*_Proc_ = *I*_mod_ − *I*_ctrl_; Golowasch and Marder, 1992). Proctolin currents were measured after at least 10 min of proctolin bath application.

During an ongoing pyloric rhythm, LP produces bursts of spikes on top of slow-wave oscillations (Fig. 1*A*,*B*) with periods typically ranging from 0.5 to 2 Hz across experiments. We approximated LP’s depolarization rate at different cycle frequencies as linear ramps (Fig. 1*C*) with slopes ranging from 50 mV/s to 400 mV/s (Extended Data Fig. 1-1).

To measure the *I-V* relationship of proctolin currents, we voltage clamped the LP neuron at −80 mV and ramped its voltage symmetrically from −80 to +20 mV [positive (+) ramp] and back to −80 mV [negative (–) ramp] with different slopes (Swensen and Marder, 2000; Garcia et al., 2015; Fig. 1*D*, left). We repeated each triangular ramp five times with 5 s intervals in between, during which time the cell was held at −80 mV. In these measurements, we assumed fast activation of the proctolin currents (Golowasch and Marder, 1992), and assumed that the current measured in the negative ramp excluded currents that inactivated during the positive ramp. Since the proctolin current is a difference current, unmodulated currents are removed by the subtraction and do not contribute to either of the thus measured currents on the positive or negative ramps. To measure the proctolin-activated current in the absence of Ca^2+^ as done previously (Golowasch and Marder, 1992), we blocked Ca^2+^ with 200 μm Cd^2+^ (Sigma-Aldrich), added to the saline both in control and in the presence of proctolin.

To ensure complete exclusion of the inactivating proctolin currents during the negative ramp *I-V* relationship, in some experiments we used the same voltage range and slopes as before but held the voltage at +20 mV between the positive and negative ramps. Additionally, these ramp-and-hold waveforms were repeated until the *I-V* relationships reached a steady state. Thus, in these experiments the positive and negative ramps each contributed to 25% of one stimulus cycle; after the positive ramp, the voltage was held at +20 mV for 25% of the cycle, and after the negative ramp at −80 mV for 25% of the cycle, and this waveform scaled with ramp slope (Fig. 1*D*, right). Each ramp-and-hold stimulus was repeated 30 times.

We also measured *I*_Proc_ with realistic LP neuron voltage waveforms. A realistic unitary waveform was obtained by recording a typical LP neuron cycle of activity, averaging 10 cycles, and scaling the voltage so that the slow-wave amplitude ranged from −60 to −20 mV (Fig. 1*B*). The unitary waveform was applied periodically (for 20 cycles with cycle periods between 250 ms and 2 s, depolarization rates are listed in Extended Data Fig. 1-1) to the voltage-clamped LP neuron in control and proctolin-containing saline. The same realistic unitary waveform was used in all experiments.

Data were discarded if the LP neuron input resistance dropped below 5 MΩ during the experiment, if electrode offset was greater than ±5 mV at the end of the experiment, or if no inward difference current was present at the peak of the realistic waveforms.

### Analysis

**Figure 2.**
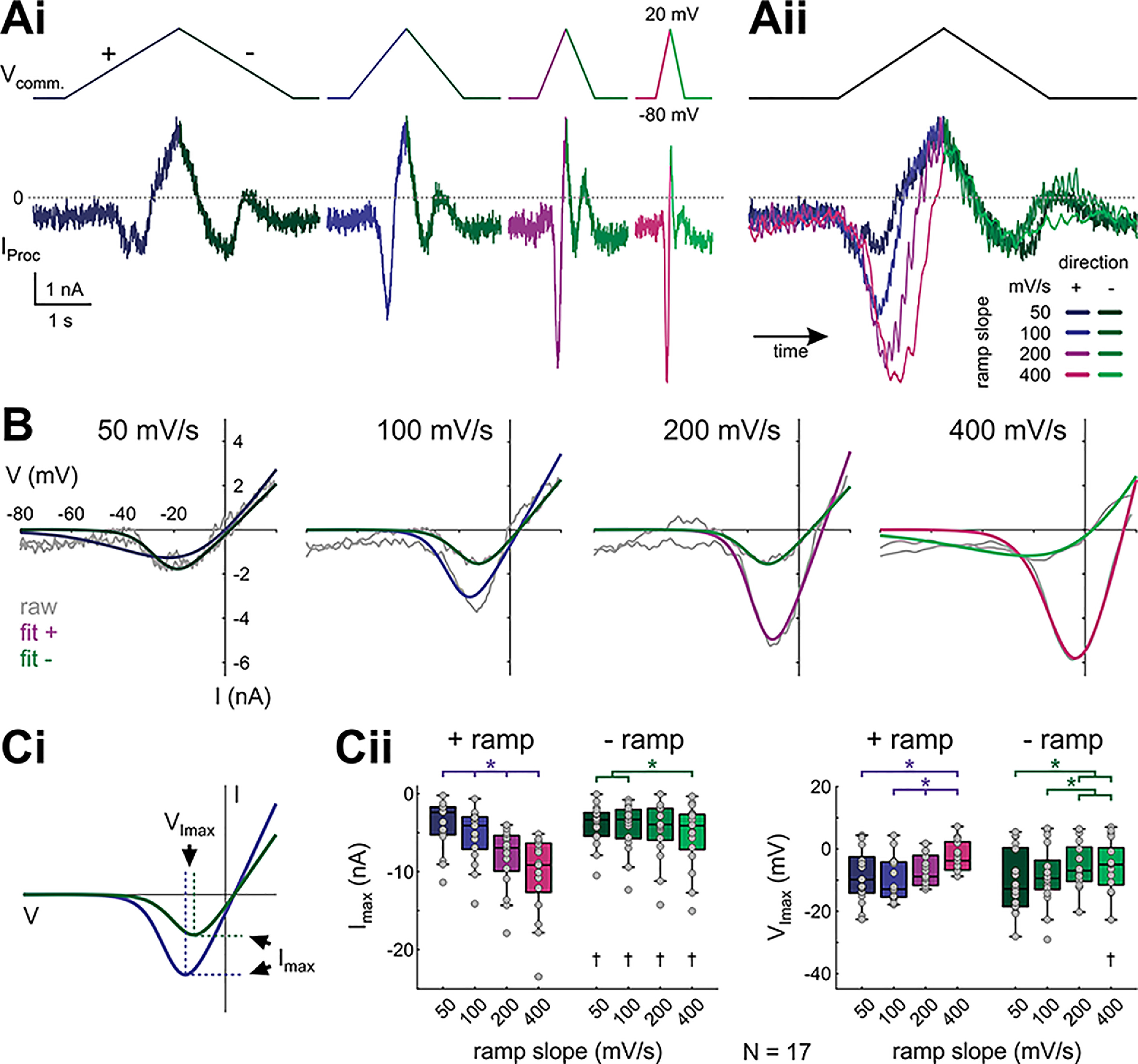
Proctolin-activated currents depend on ramp slope and direction. ***Ai***, Proctolin-activated currents (*I*_Proc_) evoked by symmetrical ramp stimulations with four different slopes (color-coded for ramp steepness and ramp direction), averaged over the last three (out of five) sweeps from one experiment. ***Aii***, Overlay of the proctolin currents shown in ***Ai***, normalized by time. ***B***, *I-V* curves for *I*_Proc_ shown in ***A***, separated by ramp slope. Gray curves show raw recordings, colored curves show logistic fits that were used to smoothe the raw data. ***C***, Quantitative analysis of the peak inward current *I*_max_ (***Cii***, left, indicated by dashed horizontal lines in ***Ci***) and voltage at peak inward current *V*_Imax_ (***Cii***, right, indicated by dashed vertical lines in ***Ci***) for different ramp slopes and ramp directions (*N* = 17). Dots represent data from individual experiments. *I*_max_ and *V*_Imax_ are both sensitive to ramp slope on the + ramp. On the – ramp, *I*_max_ and *V*_Imax_ are only significantly different between extreme slope differences (two-way RM ANOVA; Table 1; results in Extended Data Fig. 2-1). Asterisks indicate significant differences between slopes within the same direction, daggers indicate significant differences between directions within the same slope at α = 0.05.

All data were analyzed with custom written MATLAB scripts. Raw traces were first smoothed with a Savitzky–Golay filter (polynomial order: 5; frame length: 51 for *V*, 101 for *I*). Then, we subtracted the currents measured in control condition from the currents measured in the presence of proctolin to get the proctolin currents *I*_Proc_. We averaged the last three stimulation cycles for the ramp stimulations, and the last five cycles for the ramp-and-hold stimulations. We fitted the averaged *I*_Proc_ separately for the positive ramps and negative ramps with a logistic equation:

$$I_{Proc}=\frac{g\cdot (V-E_{rev})}{1+\exp\left(-\frac{V-V_{1/2}}{k}\right)},$$

where *V* is the membrane potential, *g* the maximal conductance, *E_rev_* the reversal potential, *V_½_* the half-maximal activation voltage, and k the activation slope factor. We then used these fits to identify the maximum inward current (*I*_max_) and the corresponding voltage (*V*_Imax_) at *I*_max_ at different slopes (Fig. 2*B*,*Ci*).

10.1523/ENEURO.0338-21.2021.f2-1 Extended Data Figure 2-1 Two-way RM ANOVA for initial state *I-V* parameters. α = 0.05. Direction: positive or negative. Slope (in mV/s): 50, 100, 200, 400. Data for *V*_Imax_ failed normality and equal variance tests. Download Figure 2-1, XLSX file.

10.1523/ENEURO.0338-21.2021.f2-2 Extended Data Figure 2-2 Data for panel ***Cii***. Download Figure 2-2, XLSX file.

**Table 1:** Statistical tests

Source		Data structure	Type of test	Power or [25%, 75%]
Fig. 2	*I*_max_	Normal	Two-way RM ANOVA	Sign: 1
				Speed: 1
				Interaction: 1
Fig. 2	*V*_Imax_	Non-normal	Two-way RM ANOVA	Sign: 0.05
				Speed: 1
				Interaction: 0.808
Fig. 3	Steady/initial state	Normal	RM ANOVA	0.999
Fig. 3	τ	Non-normal	ANOVA on ranks	100 mV/s: [3.3, 16.55]
				200 mV/s: [3.7, 11.98]
				400 mV/s: [3.15, 3.93]
Fig. 4	*I*_max_	Normal	Two-way RM ANOVA	Sign: 0.622
				Speed: 0.067
				Interaction: 0.449
Fig. 4	*V*_Imax_	Normal	Two-way RM ANOVA	Sign: 0.452
				Speed: 0.333
				Interaction: 0.05
Fig. 5	*I*_max_	Normal	RM ANOVA	1
Fig. 5	φ_*I*_max__	Normal	RM ANOVA	0.998
Fig. 7	*I*_max_	Normal	Two-way RM ANOVA	Sign: 0.05
				Speed: 0.05
				Interaction: 0.089
Fig. 7	*V*_Imax_	Normal	Two-way RM ANOVA	Sign: 0.878
				Speed: 0.05
				Interaction: 0.05
Fig. 8	*I*_max_	Normal	Two-way RM ANOVA	Sign: 0.709
				Speed: 0.996
				Interaction: 0.672
Fig. 8	*V*_Imax_	Normal	Two-way RM ANOVA	Sign: 0.379
				Speed: 0.206
				Interaction: 0.543
Fig. 8	Cd^2+^ slopes	Non-normal	Two-way ANOVA	Sign: 0.05
				Saline: 0.351
				Interaction: 0.068
Fig. 8	*I*_Proc(max)_	Normal	Two-way RM ANOVA	State: 0.465
				Speed: 0.446
				Interaction: 0.232

To calculate the time constant for the slow inactivation, we fitted the normalized, integrated *I*_Proc_ across the 30 ramp-and-hold sweeps with an exponential decay function:

$$I_{Proc}(t)=(I_{Proc}(t_{0})-I_{Proc}(\infty ))\cdot exp\left(-\lambda \cdot t\right)+I_{Proc}(\infty ),$$

where *t* is time and *I*_Proc_ (t_0_) and *I*_Proc_ (∞) are, respectively, the initial and steady state value of *I*_Proc_.

To measure *I*_Proc_ acquired with the realistic waveform stimulations, the current was baseline subtracted and filtered (Savitzky–Golay filter, polynomial order: 3, frame length: 901). Baseline current was measured as the average current in the 2 s before the start of stimulation. *I*_Proc_ was averaged for the last five cycles of stimulation, and we obtained *I*_max_ as well as the phase of *I*_max_ with LP trough potential as reference.

All statistical analyses were performed in SigmaPlot 12.0 (Systat Software). For parameters obtained from ramp and ramp-and-hold stimulations, we used two-way repeated measures ANOVA (RM ANOVA) to test for effects of ramp slope (in mV/s: 50, 100, 200, 400), ramp direction (positive, negative), and interaction between the two factors. For the maximum inward current and phase of the maximum inward current obtained from realistic waveform stimulations, as well as the ratio of slow inactivation across ramps, we used one-way RM ANOVA. We compared time constants of slow inactivation across ramps with one-way ANOVA on ranks. To compare changes in *I*_max_ between normal and Cd^2+^- saline we used two-way ANOVA. We used Tukey’s *post hoc* test for all pairwise comparisons. Data passed all tests for normality (Shapiro–Wilk) and equal variance (Levene’s test) unless noted otherwise. Significance level is α = 0.05. Results of statistical tests and raw data are provided in Extended Data Figures 2-1, 3-1, 3-2, 4-1, 5-1, 7-1, 8-1, 8-2, 8-3, and 2-2, 3-3, 4-2, 5-2, 7-2, 8-4, 8-5, 8-6, 9-1, respectively. An overview of the statistical tests is provided in Table 1.

### Modeling the voltage-clamp currents

The previously identified persistent current activated by proctolin has been found to be the same current as the persistent inward current activated by other modulators (Golowasch and Marder, 1992; Swensen and Marder, 2000) and thus later named the modulator-activated inward current, *I*_MI_. Here, the steady-state *I*_Proc_ was modeled as a sum of two currents, the persistent current *I*_MI_ and a transient current *I*_MI-T_:

$$\begin{array}{c} I_{Proc}=I_{MI}+I_{MI-T}\\ I_{MI}={\bar{g}}_{MI}m_{MI}(V-E_{MI})\\ I_{MI-T}={\bar{g}}_{MI-T}m_{MI-T}^{3}h_{MI-T}(V-E_{MI-T}) \end{array}$$

where the kinetic variables (*m*_MI_, *m*_MI-T_ and *h*_MI-T_—denoted as *x*) obeyed standard Hodgkin–Huxley style kinetics:

(1) $$\frac{dx}{dt}=\frac{x_{\infty }(V)-x}{\tau_{x}(V)}.$$

The parameters for the model are provided in Table 2. The slowly decaying current was modeled by modeling *I*_MI-T_ as a (putative) calcium current using the Goldman–Hodgkin–Katz formalism:

(2) $$\begin{array}{c} I_{Ca}=P_{Ca}m_{Ca}^{3}h_{Ca}F\zeta \left(\frac{{\left[Ca\right]}_{out}e^{-\zeta }-{\left[Ca\right]}_{in}}{e^{-\zeta }-1}\right)\\ \zeta =\frac{z_{Ca}vol\cdot F}{RT}V \end{array}$$

where *P*_Ca_ is the total permeability of the current, *m*_Ca_ and *h*_Ca_ are activation and inactivation variables obeying Equation 1, *vol* is the volume of the microdomain influencing the current, *F* is Faraday’s constant, *R* is the universal gas constant and *T* is temperature. [*Ca*] is the calcium concentration outside (*out*) and inside (*in*) the cell. The internal calcium concentration obeyed the equation

(3) $$\frac{d{\left[Ca\right]}_{in}}{dt}=\frac{{\left[Ca\right]}_{\infty }-{\left[Ca\right]}_{in}}{\tau_{Ca}}-\frac{P_{1}}{z_{Ca}F\cdot vol\cdot P}I_{Ca},$$

where *I_Ca_* denotes the total calcium current flowing into the cell (in this case, *I*_Ca_ = *I*_MI-T_) and [*Ca*]_∞_ denotes the steady state calcium concentration inside the cell, and *P*_1_ is the maximal per cluster permeability of *I_Ca_* and *P* is the total permeability over all clusters of interest (Taylor et al., 2009). The parameters of *I*_MI-T(Ca)_ were *P*_Ca_ = 0.014 cm/s, *T *=* *283.15 K, [*Ca*]_out_ = 13 mm, *P*_1_ = 1.1675 μm^3^/s, *P *=* *0.0369 cm/s, vol = 6.49 μm^3^, [*Ca*]_∞_ = 0.02 mm, *h*_∞_([*Ca*]) = 1/(1+([*Ca*]/0.015)^4^) and τ_Ca_ = 12 s.

**Table 2:** Parameters of the steady-state model of the proctolin-activated current

Variable/parameter	Value
$\bar{g}$_MI_, $\bar{g}$_MI-T_ (nS)	0.125, 1.00
*E*_MI_, *E*_MI-T_ (mV)	35, 24
*m*_MI_*_∞_*(*V*)	$S(\frac{v+19}{18})$
*τ*_mMI_ *(V)*	2
*m*_MI-T∞_(*V*)	$S(\frac{v+11.8}{12})$
*τ*_mMI-T_ *(V)*	$2+\frac{150}{\cosh\left(\frac{v+20}{4}\right)}$
*h*_MI-T∞_(*V*)	$S(-\frac{v+40}{7})$
*τ*_hMI-T_ *(V)*	100+600cosh(0.1(v + 30))+ 200(0.2(v + 30))

In this table, S(*x*) denotes the logistic sigmoid function 1/(1+exp(–*x*)). Time constants are in milliseconds.

### The LP model neuron

#### LP model implementation

An LP inspired model was adapted from Taylor et al. (2009), reduced to two compartments: a soma/neurite and an axon. We built this model explicitly as a two-compartment model rather than using neuron’s in-built compartmentalization process. The two compartments were each built to have a diameter and length of 200 μm and were coupled with a conductance of 0.15 μS. The soma/neurite currents included were *I*_leak_, *I*_A_, *I*_h_, *I*_Ca_, *I*_K(Ca)_, *I*_MI_, and *I*_MI-T_. Intracellular calcium accumulation was tracked in the soma/neurite compartment according to Equation 3. The ionic currents included in the axon were *I*_leak_, *I*_Na_, and *I*_K_. Except for *I*_Ca_, all currents were modeled in the standard Hodgkin–Huxley formalism as:

$$I_{x}={\bar{g}}_{x}m_{x}^{a}h_{x}^{b}(V-E_{x}),$$

where *X* denotes the current name, *E_x_* is the reversal potential and *m_x_* and *h_x_* respectively denote the current activation and inactivation (obeying Eq. 1) with appropriate powers, *a* and *b*. *I*_Ca_ was modeled as in Equation 2. The reversal potentials (in mV) for the ionic currents were set to *E*_Na_ = 50, *E*_K_ = *E*_A_ = −80, *E*_h_ = −10, *E*_leak_ = −55, *E*_MI_ = −10, *E*_MI-T_ = −16. The maximal conductances (in μS/mm^2^) were set to ${\bar{g}}_{Na}=59.68$, ${\bar{g}}_{K}=17.90$, $g_{leak,Axon}=0.199$, ${\bar{g}}_{A}=0.059$, ${\bar{g}}_{h}=0.318$, ${\bar{g}}_{K(Ca)}=0.159$, ${\bar{g}}_{MI}=0.283$, ${\bar{g}}_{MI-T}=2.83$, and $g_{leak,S/N}=0.795$. The values of ${\bar{g}}_{MI}$ and ${\bar{g}}_{MI-T}$ were adjusted as described in the figure legends. The membrane capacitance (in μF/cm^2^) was set to 0.795 (axon) and 3.183 (soma/neurite). The parameters of *I*_Ca_ were *P*_Ca_ = 0.00187 nm/ms, *T *=* *283.15 K, [*Ca*]_out_ = 13 mm, *P*_1_ = 1.168 μm^3^/s, *P *=* *0.0467 nm/ms, vol = 6.49 μm^3^. The equations for the ionic currents are given in Table 3.

**Table 3:** LP neuron model equations

		*m*_∞_	*τ_m_*	*h*_∞_	*τ_h_*	Compartment
*I*_A_	*m* ^2^ *h*	$\text{S}(\frac{v+54}{10})$	15	$\text{S}(-\frac{v+60}{5})$	100	Soma/neurite
*I*_Ca_	*m* ^2^ *h*	$\text{S}(\frac{v+45}{15})$	$2+2\text{S}\left(\frac{v+40}{20}\right)$	$\text{S}(-\frac{{\left[Ca\right]}_{in}}{12.57})$	100	Soma/neurite
*I*_h_	*m* ^2^	$\tanh^{+}\left(\frac{v+58}{10}\right)$	200	-	_-_	Soma/neurite
*I*_K(Ca)_	*mh*	$\frac{\text{S}(\frac{v+45.5}{2})}{1+\frac{0.00715}{{\left[Ca\right]}_{in}}}$	100	$\frac{1}{1+{\left(\frac{{\left[Ca\right]}_{in}}{0.03}\right)}^{1.25}}$	10	Soma/neurite
*I*_MI_	*m*	$\text{S}\left(\frac{v+19}{18}\right)$	2	-	-	
*I*_MI-T_	*m* ^3^ *h*	$\text{S}\left(\frac{v+52.5}{4.8}\right)$	$2+\frac{150}{\cosh\left(\frac{v+56}{1.6}\right)}$	${\left[\text{S}\left(-\frac{v+61}{2.8}\right)-0.1\right]}^{+}$	100+600cosh(0.25(v+60))+200S(0.5(v+60))	Soma/neurite
*I*_Na_	*m* ^3^ *h*	$\text{S}\left(\frac{v+18}{12.25}\right)$	2	$\text{S}\left(-\frac{v+28}{7.7}\right)$	5	Axon
*I*_K_	*m* ^4^	$\text{S}\left(\frac{v+23}{5}\right)$	$2+14\text{S}\left(-\frac{v+23}{5}\right)$	-	-	Axon

In this table, S(*x*) denotes the logistic sigmoid function 1/(1+exp(–*x*)), *f*^+^(*x*) denotes the positive part of *f*(*x*) (with the negative portions set to 0), voltage (*v*) is in mV, intracellular calcium concentration ([*Ca*]_in_) is in millimolar, and time constants (*τ_m_*, *τ_h_*) are in milliseconds.

Inhibitory synaptic input was modeled as a symmetric triangular conductance waveform with a duty cycle of 0.5, a maximum value of 1 μS/mm^2^ and a reversal potential of −80 mV. The model was then manually tuned to produce an LP-like voltage waveform.

#### Frequency effects on the model LP neuron

Modulator receptors are thought to be localized far away from the soma (Golowasch and Marder, 1992). It is reasonable to assume that, in a voltage-clamp experiment, voltages in distal compartments are different from the somatic clamped voltage. To account for this, we shifted the voltage dependencies of equations describing the parameters of *I*_MI-T_ in our model LP neuron. This shift was estimated by adjusting the model for the steady state ramp-and-hold to respond as if *I*_MI_ and *I*_MI-T_ were expressed in a compartment ∼0.3 length constants away from the soma, rather than in the soma (fitting not shown). All simulations of the model LP neuron were done with these shifted values (Table 3).

To examine the frequency-dependent effects of *I*_MI_ and *I*_MI-T_ versus *I*_MI_ or *I*_MI-T_ alone, we built three different models and matched the spike numbers in all three models at a cycle frequency of 1 Hz. We then modified the cycle frequency of the synaptic input in each model and examined the effect of the cycle frequency in each case. Activity was simulated for 20 cycles to reach a steady state, with only the last four cycles considered for analysis. The bursting attributes we measured were phase (relative to the peak of the synaptic conductance input) and instantaneous spike frequency (defined as the reciprocal of the inter-spike interval). Simulations were done in the Python NEURON (version 7.7.2) environment (Carnevale and Hines, 2006), and analyses were performed using custom Python scripts.

The code described in the paper is freely available online at https://github.com/fnadim/IMI-T and also as the Extended Data 1.

10.1523/ENEURO.0338-21.2021.ed1 Extended Data 1 The codes used in the study. Download Extended Data 1, ZIP file.

## Results

Previous measurements of peptide neuromodulation of pyloric neurons demonstrated that multiple peptides activate a fast voltage-dependent persistent inward current, *I*_MI_ (Swensen and Marder, 2000), which was first characterized as a proctolin-activated current in the LP neuron (Golowasch and Marder, 1992). Here, we explored the possibility that modulatory neuropeptides may activate additional currents in pyloric neurons that would allow their modulatory effect to be sensitive to the frequency of oscillations and thus increase the dynamic range of neuromodulatory responses of these cells.

### The amplitude of *I*_Proc_ is sensitive to the slope and direction of voltage ramps

During an ongoing pyloric rhythm, the LP neuron membrane potential expresses slow-wave oscillations in time with the pyloric rhythm (Fig. 1*A*). We approximated the LP neuron waveform (Fig. 1*B*) slow-wave depolarization slope at different cycle periods (Fig. 1*C*) and measured the proctolin-activated current *I*_Proc_ with ramp voltage protocols with corresponding slopes (Fig. 1*D*; Extended Data Fig. 1-1; see Materials and Methods).

With both positive and negative voltage ramps, proctolin elicited a voltage-gated inward current (*I*_Proc_), which had a similar appearance for all ramp slopes (Fig. 2*A*). However, the maximum magnitude (*I*_max_) of *I*_Proc_ was larger on the positive ramp than on the negative ramp. Furthermore, the magnitude of the current was sensitive to the slope of the positive ramp, with larger slopes eliciting larger currents (Fig. 2, raw difference currents of one example experiment in *A*, corresponding *I-V* curves with fits in *B*, quantification in *C*). For the negative ramp, *I*_max_ was mostly independent of slope value, except for the largest slope of 400 mV/s. Additionally, the voltage (*V*_Imax_) at which *I*_max_ was attained was more depolarized for larger slopes on the positive ramp, and less so on the negative ramp (Fig. 2*C*; two-way RM ANOVA results in Extended Data Fig. 2-1). Together, these data suggested that *I*_Proc_ includes both a persistent and a transient (inactivating) component. Both components activated with the positive ramp, but the negative ramp elicited mostly the persistent component because, by this time, the other component had inactivated. The presence of these two components would explain (1) the difference between the current amplitudes on the positive and negative ramps; (2) the slope dependence of the current amplitude on the positive ramps; (3) the fact that the largest negative ramp slope also revealed a larger current compared with other negative ramps, likely because of incomplete inactivation of the transient component; and (4) the shift of *V*_Imax_ to more depolarized values for larger slopes because with these more depolarized voltages are reached in a shorter amount of time, before complete inactivation of the transient component.

The component of *I*_Proc_ measured with negative ramps was (mostly) independent of ramp slope and qualitatively matched the previously described *I*_MI_ (Golowasch and Marder, 1992; Swensen and Marder, 2000; Goaillard et al., 2009; Gray and Golowasch, 2016). Therefore, we will refer to this persistent component as *I*_MI_, and to the inactivating (transient) component as *I*_MI-T_. Thus, the negative ramp current consists primarily of *I*_MI_, whereas the positive ramp current consists of *I*_MI_ + *I*_MI-T_.

### *I*_Proc_ has an additional, slow inactivating component

**Figure 3.**
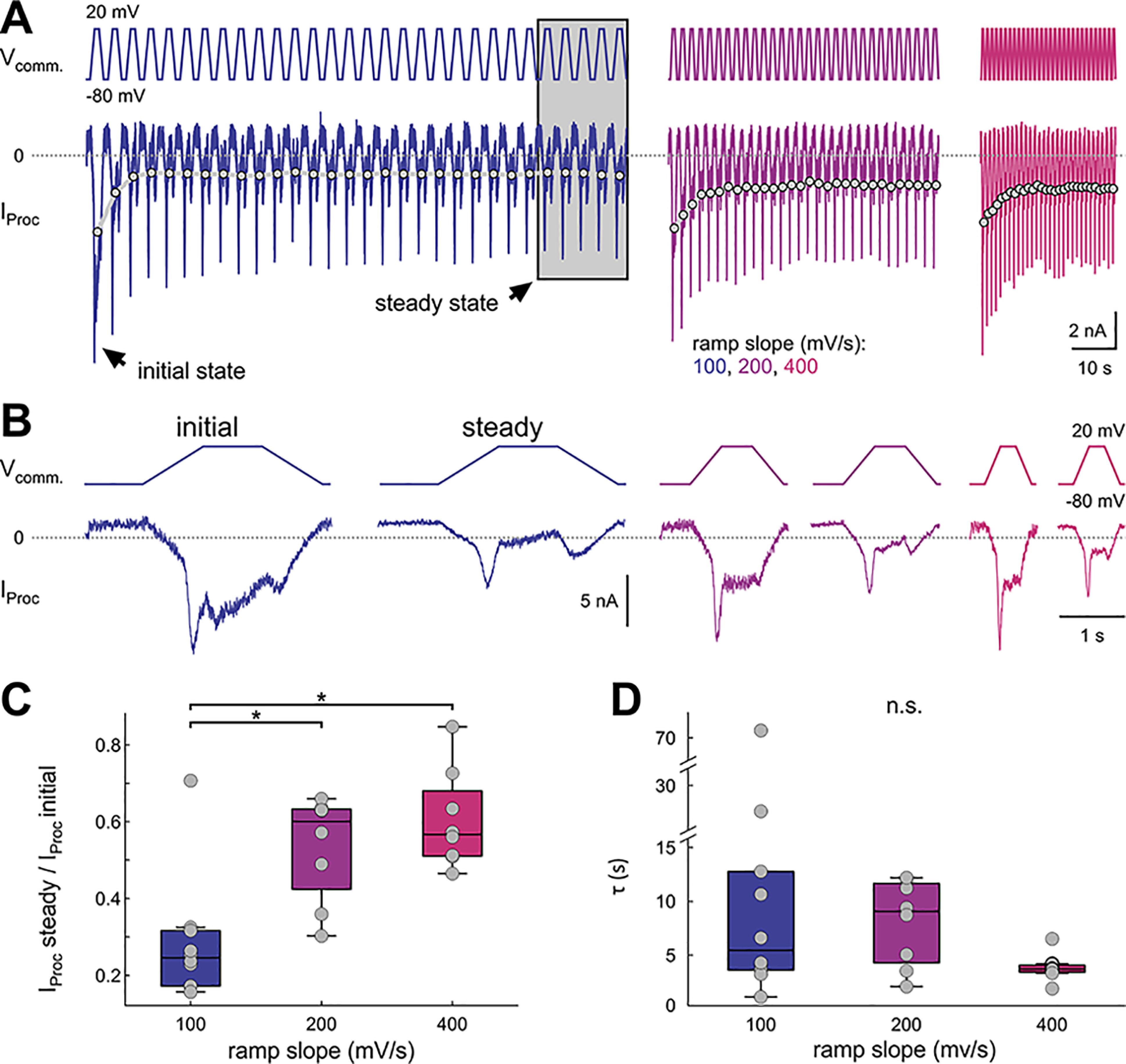
Proctolin-activated currents show slow inactivation. ***A***, Proctolin-activated currents (*I*_Proc_) in response to 30 sweeps of ramp-and-hold stimuli with different slopes (color-coded). The gray dots connected by lines depict the average *I*_Proc_ for each sweep in this experiment. We refer to *I*_Proc_ during the first sweep as initial state, and to the average of the last five sweeps as steady state (gray box). ***B***, First (initial state) and averaged last five (steady state) sweeps from the experiment in ***A***. ***C***, Ratio of *I*_Proc_ between steady state and initial state. The slow inactivation of *I*_Proc_ is greater at 100 mV/s ramps compared with 200 and 400 mV/s ramps (RM ANOVA; Table 1; results in Extended Data Fig. 3-1). Each dot represents an individual experiment. ***D***, Time constants for slow inactivation. Each dot represents an individual experiment. Time constants were not significantly different between slopes (ANOVA on ranks; Table 1; results in Extended Data Fig. 3-2). Asterisks indicate significance at α = 0.05. n.s. indicates no significant changes.

In the ramp measurements (Fig. 2), we allowed a 5 s time interval between ramps, so that the currents measured could recover from any inactivation. Hence, there was little difference between currents in subsequent identical ramp measurements. However, in normal biological conditions, the pyloric rhythm is active continuously and, therefore, *I*_Proc_ would be at some steady-state level of inactivation. To measure the steady-state levels of *I*_MI_ and *I*_MI-T_, we applied symmetric ramp-and-hold stimuli repetitively for 30 cycles in a separate set of experiments (Fig. 3*A*). We switched to a ramp-and-hold protocol, expecting that the addition of a depolarized hold interval between the positive and negative ramp would allow for full inactivation of any inactivating component and, therefore, a better separation of *I*_MI_ and *I*_MI-T_.

10.1523/ENEURO.0338-21.2021.f3-1 Extended Data Figure 3-1 One-way RM ANOVA for the steady/initial state ratios of I_Proc_. α = 0.05. Slopes (in mV/s): 100, 200, 400. Missing N: 200 mV/s: 2; 400 mV/s: 2. Download Figure 3-1, XLSX file.

10.1523/ENEURO.0338-21.2021.f3-2 Extended Data Figure 3-2 One-way ANOVA on ranks for the time constant of the slow inactivation. α = 0.05. Slope (in mV/s): 100, 200, 400. Missing N: 200 mV/s: 2; 400 mV/s: 2. Download Figure 3-2, XLSX file.

10.1523/ENEURO.0338-21.2021.f3-3 Extended Data Figure 3-3 Data for panels ***C***, ***D***. Download Figure 3-3, XLSX file.

The repeated depolarization in this longer protocol revealed a clear slow reduction in the amplitude of the total current (Fig. 3; raw difference currents across all sweeps of one experiment in *A*, initial and steady state sweeps of the same experiment in *B*, quantification of the levels of slow inactivation in *C* and of the time course in *D*). We refer to the response to the first depolarizing ramp-and-hold stimulus in each run as the initial state, and to the average of the last five responses as steady state (Fig. 3*A*,*B*). The distinction between the positive and negative ramp currents was present both in the initial state and at steady state (Fig. 3*A*,*B*). The first stimulus produced a current that was comparable to the currents we measured with triangular ramps and analyzed in Figure 2, and therefore we do not repeat that analysis here. To quantify the slow change, we measured the average inward current amplitude during each stimulus (Fig. 3*A*, gray dots connected by lines). A comparison of the initial response amplitude with that at steady state showed a slope-dependent reduction in amplitude for all ramp slopes (Fig. 3C; statistical comparisons in Extended Data Fig. 3-1). Note that this reduction in amplitude was very slow, in the range of several seconds (Fig. 3*D*) and therefore distinct from the fast inactivation effect that distinguished the positive and negative ramp currents. The time constant of the reduction of the current amplitude at this slow timescale did not depend on the steepness of the ramp slope (ANOVA on ranks; results in Extended Data Fig. 3-2).

**Figure 4.**
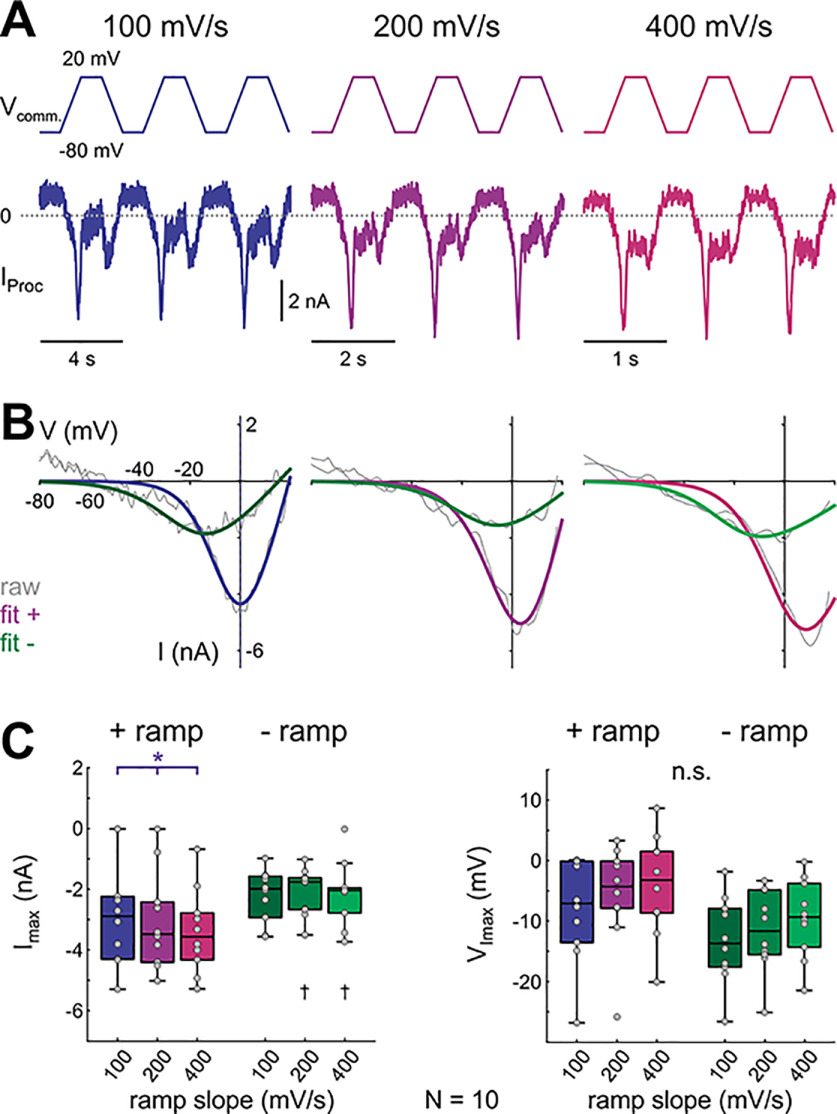
Steady-state levels of the proctolin-activated currents elicited by a periodic ramp-and-hold stimulus depend on ramp slope and direction. ***A***, Proctolin-activated currents (*I*_Proc_) in response to the last three of 30 sweeps of ramp-and-hold stimuli with different slopes (color-coded). Data are from the same experiments as Figure 3*A*. ***B***, Steady-state *I-V* curves for different ramp slopes (color-coded) from one experiment (same experiment as in ***A***). Gray lines show raw current recordings, colored lines show logistic fits that were used to smoothe the raw data. ***C***, Quantitative analysis of *I*_max_ (left) and *V*_Imax_ (right) for different ramp slopes and ramp directions (*N* = 10). Dots represent data from individual experiments. *I*_max_ is sensitive to ramp slope on the + ramp but not to the – ramp (two-way RM ANOVA; Table 1; results in Extended Data Fig. 4-1). Asterisks indicate significant differences between slopes within the same direction, daggers indicate significant differences between directions within the same slope at α = 0.05. n.s. indicates no significant changes.

We explored the effect of ramp slopes at steady state (Fig. 4, raw difference currents of three steady state cycles of one experiment in *A*, corresponding *I-V* curves with fits in *B*, quantification in *C*) as in the triangular ramp experiments without a depolarized hold interval. Generally, the steady-state *I-V* curves showed the same slope and directional dependencies as in the simple ramp currents described in Figure 2 (Fig. 4*B*,*C*; two-way RM ANOVA results in Extended Data Fig. 4-1), with one exception: *I*_max_ and *V*_Imax_ values at steady state on the negative ramp no longer showed slope dependence, probably because this difference was due to incomplete inactivation of the transient component with the triangular ramps.

10.1523/ENEURO.0338-21.2021.f4-1 Extended Data Figure 4-1 Two-way RM ANOVA for steady state *I-V* parameters (ramp-and-hold). α = 0.05. Direction: positive or negative. Slope (in mV/s): 100, 200, 400. Download Figure 4-1, XLSX file.

10.1523/ENEURO.0338-21.2021.f4-2 Extended Data Figure 4-2 Data for panel ***C***. Download Figure 4-2, XLSX file.

Together, these findings led to two conclusions. First, *I*_Proc_ has a slowly inactivating component, with a time constant of several seconds, that diminishes the total current activated and measured across frequently repeated stimuli until it reaches stable levels at steady state. Second, even at steady state, *I*_Proc_ has a persistent component (*I*_MI_) and a component that inactivates rapidly during depolarizing ramps (*I*_MI-T_).

### The amplitude of *I*_Proc_ is sensitive to the cycle period of the pyloric rhythm

**Figure 5.**
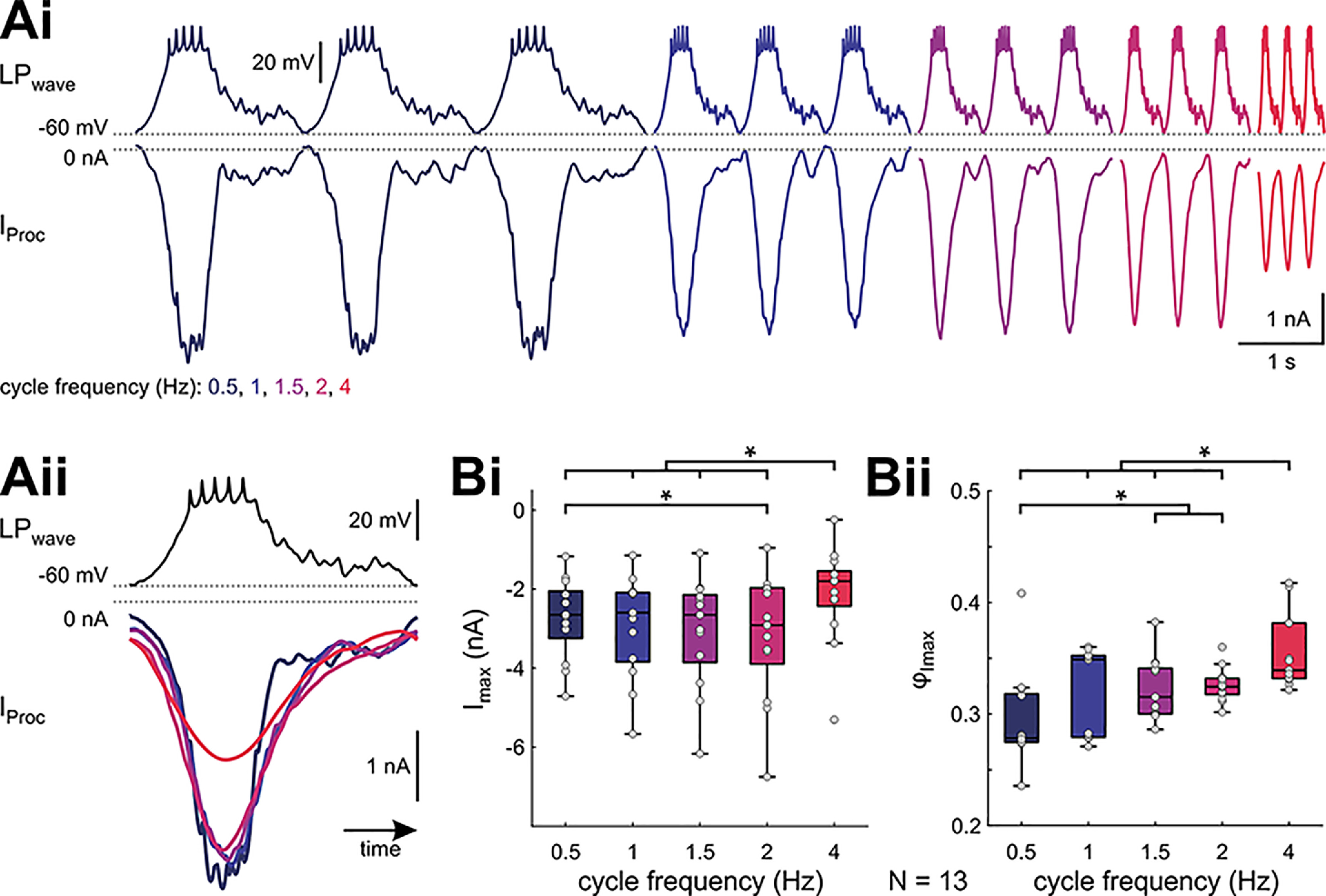
Slope-sensitivity of proctolin-activated currents is different during ongoing LP activity. ***Ai***, The last three (of 20) sweeps of the proctolin-activated currents *I*_Proc_ in response to voltage clamping with a realistic LP waveform. Shown is one experiment at different cycle frequencies (color-coded). ***Aii***, Overlay of the averages of the last five cycles (same experiment as ***Ai***), normalized to time. ***B***, *I*_max_ and phase of *I*_max_ (ϕ_Imax_) are sensitive to cycle frequency (RM ANOVA; Table 1; results in Extended Data Fig. 5-1). Dots represent data from individual experiments. Asterisks indicate significant differences between frequencies at α = 0.05.

The *I-V* curves revealed that *I*_Proc_ was most sensitive to the depolarizing slope. Because the pyloric rhythm operates over a large range of cycle periods (Bucher et al., 2005), and the shape of the voltage waveform (e.g., rising and falling slopes) is influenced by this period, the size of the inward current activated by proctolin would also be expected to be influenced by the period. We, therefore, measured how the cycle period of the LP waveform influenced the total current activated by proctolin. To do so, we repeatedly played back a prerecorded LP voltage waveform scaled to, and applied at, different cycle periods in the voltage-clamped LP neuron, in control and in the presence of proctolin, and measured *I*_Proc_ as the difference current (Fig. 5*A*). We used periods of 250–2000 ms (4–0.5 Hz), which corresponds to rates of membrane potential rise of ∼100–950 mV/s (see Fig. 1*C*; Extended Data Fig. 1-1).

10.1523/ENEURO.0338-21.2021.f5-1 Extended Data Figure 5-1 RM ANOVA for parameters obtained with realistic waveform stimulation. Download Figure 5-1, XLSX file.

10.1523/ENEURO.0338-21.2021.f5-2 Extended Data Figure 5-2 Data for panel ***B***. Download Figure 5-2, XLSX file.

Based on our ramp and ramp-and-hold measurements we expected that *I*_Proc_ would increase with increasing waveform cycle frequency (or shorter cycle period), which corresponds to an increase in its depolarization slope. There was a small increase in the average *I*_Proc_ amplitude as cycle frequency was increased up to 2 Hz (Fig. 5*Bi*), but this increase was not observed in every experiment (Fig. 5*Aii*). Surprisingly, and contrary to our expectation from the ramp measurements, the peak *I*_Proc_ value (*I*_max_) was significantly smaller for the highest frequency tested (4 Hz; Fig. 5*Bi*; RM ANOVA results in Extended Data Fig. 5-1). Finally, the phase (peak time relative to the trough potential divided by period) at which *I*_max_ was measured at each period got slightly but significantly delayed at the higher cycle frequencies (Fig. 5*Bii*).

### *I*_MI-T_ explains the observed frequency dependence of proctolin effects

Our findings so far led to three observations on *I*_Proc_ in the LP neuron. (1) Ramps activate a current whose amplitude depends on the steepness and sign of the slope. (2) Realistic waveforms produce a current whose amplitude somewhat increases with increasing cycle frequencies (and thus larger positive slopes) up to 2 Hz but decreases at the highest frequency tested (4 Hz) in contrast to what is predicted from the ramp experiments. (3) The size of the current decreases gradually to a steady state over a timescale of several seconds when stimuli are repeated at frequencies consistent with pyloric cycle frequencies.

**Figure 6.**
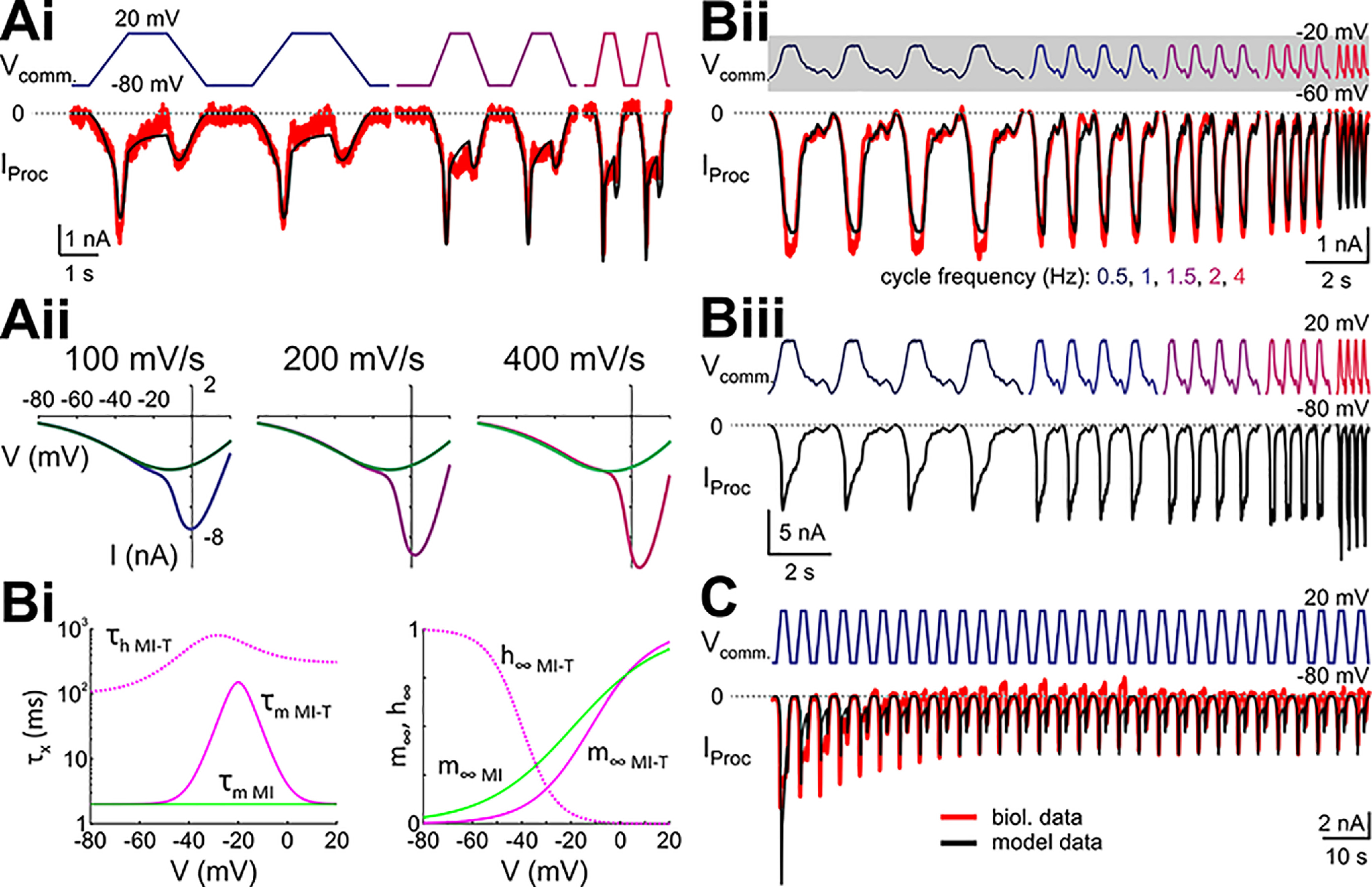
A model with only *I*_MI_ and *I*_MI-T_ adequately captures the fast and slow inactivations. ***A***, Model parameters for *I*_MI_ and *I*_MI-T_ were tuned to capture the steady-state *I*_Proc_ trajectories, i.e., a larger inward current on the positive ramps, and larger inward currents with larger slopes. ***Ai***, Overlay of the biological (red) and model (black) *I*_Proc_ trajectories in response to steady-state ramp-and-hold stimuli. ***Aii***, *I-V* curves separated by positive (purple) and negative (green) ramps similar to those shown in ***Ai***. *I-V* curves for the positive ramps were obtained after holding the voltage at –80 mV to remove inactivation, and the negative ramps after holding the voltage at +20 mV to maximize inactivation of the transient current. ***Bi***, Time constants for the activation (solid lines) and inactivation (dashed lines) gates of the model *I*_MI_ (green) and *I*_MI-T_ (pink). ***Bii***, Model response to realistic LP waveform stimulations with different cycle frequencies (based on the biological data from the same preparation as in ***Ai***). The gray shading around V_comm_. indicates the voltage range of the ramp and ramp-and-hold stimuli used in ***Ai***. ***Biii***, Model response to the same waveforms as in Bii but with an upscaled amplitude that is similar to the amplitude of the ramp and ramp-and-hold stimuli. ***C***, The slow inactivation of the proctolin-activated current can be mimicked in a computational model by modeling *I*_MI-T_ as Ca^2+^ current following the Goldman–Hodgkin–Katz formalism.

To see how these observations could be explained by proctolin activating voltage-dependent currents, we resorted to computational modeling. We first examined the steady-state effects of the ramp-and-hold protocols. We found that the steady-state slope-dependent effects of the data could be explained assuming that proctolin activated two voltage-gated currents (Fig. 6*A*). This match required that at least one of the two currents (which we have referred to as *I*_MI-T_) be an inactivating current but could not be explained with a single persistent inward current that has been previously described (Golowasch and Marder, 1992; Swensen et al., 2000).

However, a simple division of *I*_Proc_ into a persistent and an inactivating component did not explain the second observation. As described above, the measurement of *I*_Proc_ using realistic LP neuron voltage waveform protocols (slow-wave oscillation range −60 to −20 mV) showed that the amplitude of the current did not consistently follow the same slope dependence as the current using the ramp protocols. Rather, the value of *I*_Proc_ decreased at the largest applied cycle frequency of 4 Hz (largest depolarization slope), which was the opposite of what we expected from the ramp experiments. As expected, when we simulated the simplest model that fit the ramp data with the LP voltage waveforms it produced the expected result that the current became consistently larger, not smaller, with increased cycle frequencies, and thus did not replicate the realistic waveform data (not shown). However, we noted that in our experiments the ramps spanned a voltage range (−80 to +20 mV) that was much larger than that of the realistic waveforms (−60 to −20 mV). It was therefore possible that the currents involved have different kinetics in the more restricted voltage range. Because the persistent current *I*_MI_ has very fast activation kinetics (Golowasch and Marder, 1992), we focused on the kinetics of the inactivating current *I*_MI-T_. We found that an adjustment of the kinetics of *I*_MI-T_ to have slower activation over the voltage range of the realistic waveform but remain fast outside of this range (Fig. 6*Bi*, pink trace), was sufficient to reproduce the effect of the realistic waveform in that increasing cycle frequency would lead to a smaller *I*_Proc_ (Fig. 6*Bii*). A prediction of this modified model would be that applying the realistic waveform in the larger range of −80 to +20 mV should produce an increase of *I*_Proc_ when cycle frequency is increased. Indeed, this was precisely what we observed in the model (Fig. 6*Biii*).

So far, we focused on describing how the model currents matched the slope-dependent effects of *I*_Proc_ at steady state, both for ramps and for realistic waveforms. However, these currents could not reproduce the third observation, that the total current decayed slowly when the ramp-and-hold protocol was applied repeatedly (Fig. 3). This was because both *I*_MI_ and *I*_MI-T_ had fast activation, and the inactivation time constant of the model *I*_MI-T_ was <800 ms throughout the voltage range, which did not allow the current to keep a memory over repeated voltage waveforms. It is possible that the slow decay of *I*_Proc_ over several seconds involves yet another slower inactivation component or another slow-inactivating proctolin-activated ionic current altogether. However, there is an alternative and perhaps simpler possibility. Previous studies have suggested that the peptide-activated ion channels (such as those underlying *I*_Proc_) in the STG may have calcium permeability or even be calcium currents (Zhao et al., 2011; Oh et al., 2012; Rodriguez et al., 2013; Gray et al., 2017). If *I*_MI-T_ were a calcium current, calcium entry due to this current could change local internal calcium concentrations so that the driving force of the current would be reduced over repetitive depolarizations. To examine this possibility, we modeled *I*_MI-T_ as a calcium current using the Goldman–Hodgkin–Katz formalism (see Materials and Methods). Although we did not attempt to optimize the parameters of this model (many of which remain unmeasured), we found that this formalism could sufficiently explain how the current amplitude reduced with repetitive depolarization (Fig. 6*C*).

### Examining the model predictions

**Figure 7.**
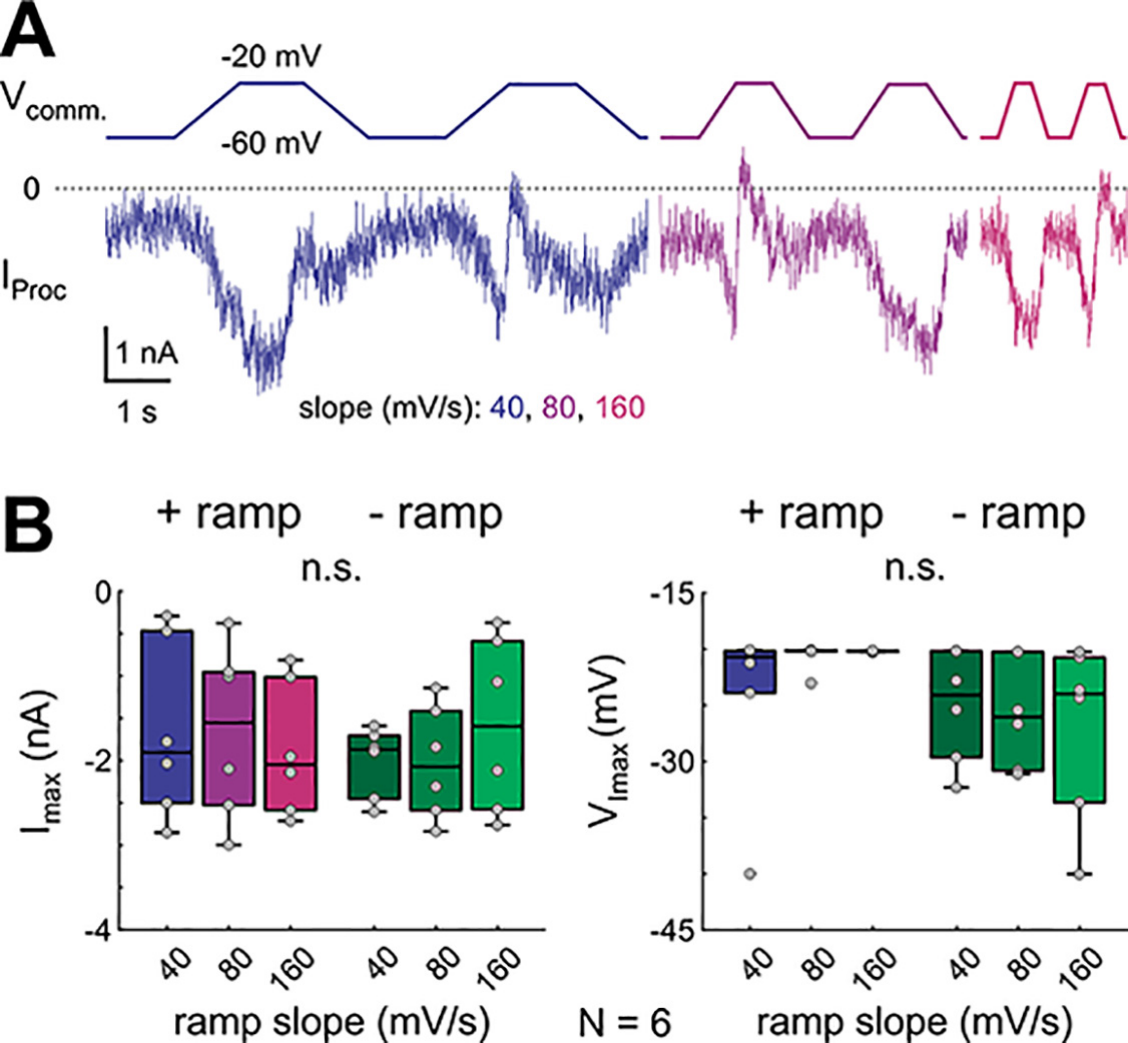
Decreasing the amplitude of ramps to that of the realistic waveforms removes the slope dependence of *I*_MI-T_. ***A***, *I*_Proc_ measured in LP in response to downscaled ramp-and-hold stimuli with an amplitude similar to the realistic LP wave stimulus. ***B***, Quantification of steady state *I*_max_ and *V*_Imax_ for the downscaled ramp-and-hold stimuli (*N* = 6). Dots represent data from individual experiments. There is no significant slope dependence (n.s.; two-way RM ANOVA; Table 1; results in Extended Data Fig. 7-1).

To examine the prediction of the model that low-amplitude stimuli produce a different slope-dependent or frequency-dependent effect than high-amplitude stimuli (Fig. 6*B*), we repeated the ramp-and-hold experiments but reduced the amplitude to match the original realistic waveform voltage range of −60 to −20 mV and scaled the cycle frequencies to match the pyloric frequencies commonly observed in experiments at 12°C (∼0.5−1 Hz; Tang et al., 2012; Haddad and Marder, 2018; Rosenbaum and Marder, 2018; Kushinsky et al., 2019; Fig. 7*A*). In this voltage range, *I*_Proc_ largely lost its slope dependence, both for *I*_max_ and *V*_Imax_ (Fig. 7*B*; two-way RM ANOVA results in Extended Data Fig. 7-1), as predicted by the model.

10.1523/ENEURO.0338-21.2021.f7-1 Extended Data Figure 7-1 Two-way RM ANOVA for steady state *I-V* parameters at small stimulus amplitude (ramp-and-hold). α = 0.05. Directions: positive or negative. Slope (in mV/s): 40, 80, 160. Download Figure 7-1, XLSX file.

10.1523/ENEURO.0338-21.2021.f7-2 Extended Data Figure 7-2 Data for panel ***B***. Download Figure 7-2, XLSX file.

**Figure 8.**
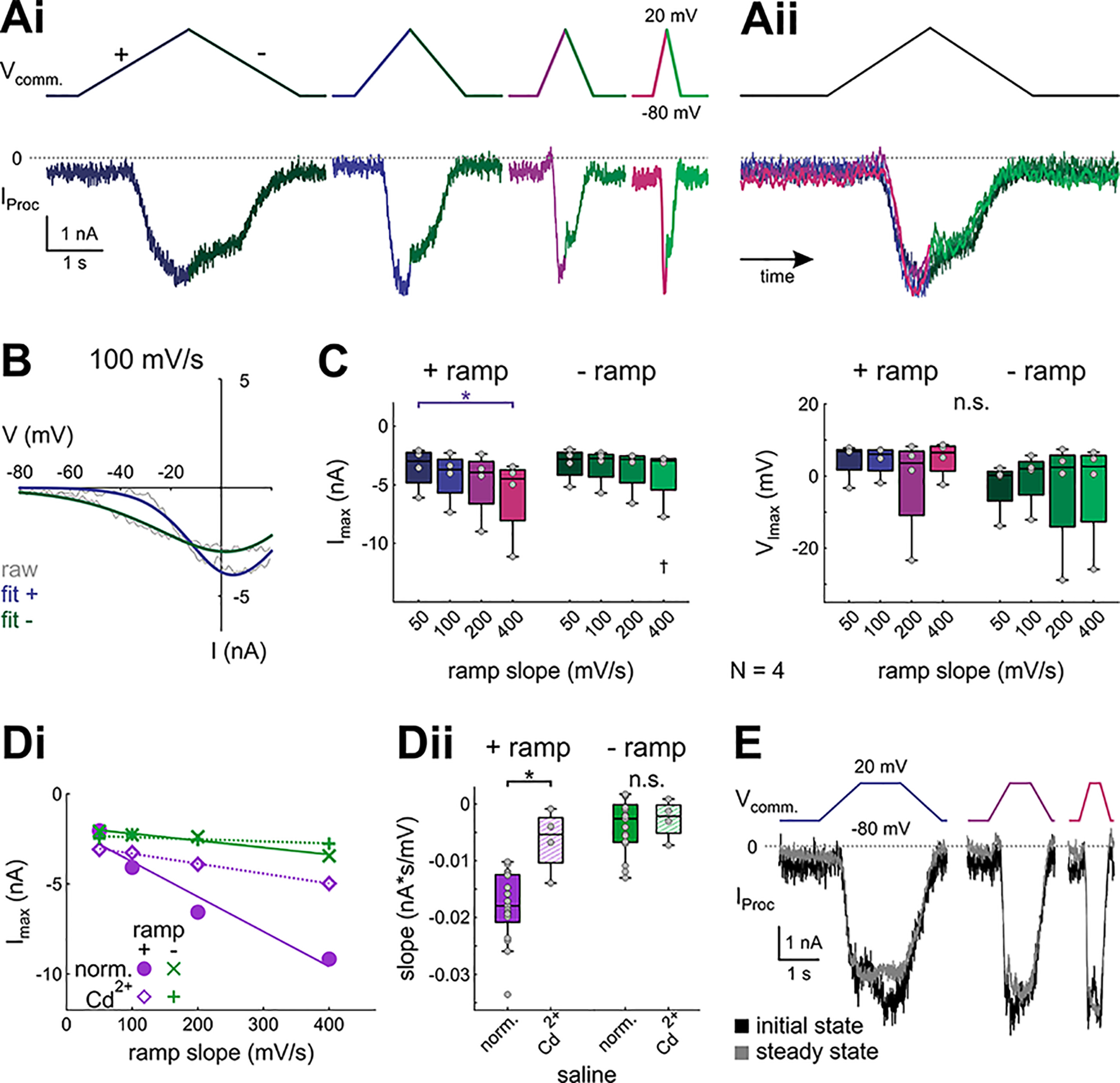
Proctolin-activated currents are partially blocked by cadmium. ***Ai***, Proctolin-activated currents in Cd^2+^ saline evoked by symmetrical ramp stimulations with four different slopes (color-coded), averaged over the last three of five sweeps of one experiment. ***Aii***, Overlay of the proctolin currents shown in ***Ai***, normalized by time. ***B***, Example *I-V* curves for *I*_proc_ shown in ***A***, 100 mV/s. Gray solid lines show original current recordings, colored solid lines show logistic fits. ***C***, Quantitative analysis of *I*_max_ (left) and *V*_Imax_ (right) for different ramp slopes and ramp directions (*N* = 4). Dots represent data from individual experiments. Ramp slope and direction show statistically significant interactions for *I*_max_ (two-way RM ANOVA; Table 1; results in Extended Data Fig. 8-1). Asterisks indicate significant differences between slopes within a direction, daggers indicate significant differences between directions within a slope at α = 0.05. n.s. indicates no significant changes. ***D***, Linear fits for *I*_max_. ***Di***, Example showing the fits from a linear regression model to the *I*_max_ for different ramp slopes and directions in normal saline and Cd^2+^ saline. ***Dii***, Slopes of linear fits for *I*_max_ in normal saline (filled boxes) and Cd^2+^ saline (hatched boxes). Cd^2+^ significantly reduced the slope of *I*_max_ for the + ramps (purple), but not the – ramps (green), indicating a reduction of *I*_MI-T_ in the presence of Cd^2+^ (two-way ANOVA; Table 1; results in Extended Data Fig. 8-2). ***E***, Example (same preparation as in ***A***) showing the initial (black) and steady-state (gray, average of the last five sweeps) responses to ramp-and-hold stimuli in Cd^2+^ saline. In all four experiments, *I*_Proc_ was not significantly different between initial and steady state (two-way RM ANOVA; Table 1; results in Extended Data Fig. 8-3).

We also tested the possibility that *I*_MI-T_ is a calcium current by repeating our ramp protocols with LP in saline containing 200 μm Cd^2+^, which blocks Ca^2+^ channels in the LP neuron (Golowasch and Marder, 1992). In Cd^2+^ saline, the slope dependence was greatly reduced (Fig. 8*A–C*, raw difference currents of one example in *A*, example *I-V* curve with fits of the same experiment in *B*, quantification in *C*). Only the largest positive slope of 400 mV/s resulted in a significantly larger *I*_max_ compared to the smallest slope of 50 mV/s (Fig. 8*C*). Additionally, except for the largest slope, *I*_max_ and *V*_Imax_ of *I*_MI_ and *I*_MI-T_ were not different in Cd^2+^ saline (Fig. 8*C*; two-way RM ANOVA results in Extended Data Fig. 8-1). In normal saline, the increase in *I*_Proc_ across positive ramp slopes was approximately linear. Therefore, we compared the slopes of linear fits across all positive and negative ramps between normal saline and Cd^2+^ saline. For the positive ramps, this slope was significantly smaller in Cd^2+^, and unchanged for the negative ramps (Fig. 8*D*; two-way ANOVA results in Extended Data Fig. 8-2), indicating that *I*_Proc_ did not depend on depolarization slope in this voltage range in Cd^2+^. We were unable to calculate time constants for the slow inactivation because *I*_Proc_ at steady-state was not different from *I*_Proc_ at the initial state in Cd^2+^ saline (Fig. 8*E*; two-way RM ANOVA; results in Extended Data Fig. 8-3). From these experiments we conclude that Ca^2+^ is at least partially involved in *I*_MI-T_, possibly as the charge carrier, and it also appears to be necessary for the slow decay of the current.

10.1523/ENEURO.0338-21.2021.f8-1 Extended Data Figure 8-1 Two-way RM ANOVA for initial state *I-V* parameters (ramps) in Cd^2+^ saline. α = 0.05. Direction: positive or negative. Slope (in mV/s): 50, 100, 200, 400. Download Figure 8-1, XLSX file.

10.1523/ENEURO.0338-21.2021.f8-2 Extended Data Figure 8-2 Two-way ANOVA for the slopes of the linear fits across ramps with the same direction. α = 0.05. Direction: positive or negative. Saline: normal or Cd^2+^. Failed normality and equal variance tests. Download Figure 8-2, XLSX file.

10.1523/ENEURO.0338-21.2021.f8-3 Extended Data Figure 8-3 Two-way RM ANOVA for *I*_Proc_ in initial and steady state in normal saline and Cd^2+^ saline. α = 0.05. State: initial or steady. Slope (in mV/s): 100, 200, 400. Download Figure 8-3, XLSX file.

10.1523/ENEURO.0338-21.2021.f8-4 Extended Data Figure 8-4 Data for panel ***C***. Download Figure 8-4, XLSX file.

10.1523/ENEURO.0338-21.2021.f8-5 Extended Data Figure 8-5 Data for panel ***Dii***. Download Figure 8-5, XLSX file.

10.1523/ENEURO.0338-21.2021.f8-6 Extended Data Figure 8-6 Data for Extended Data Figure 8-3*E*. Download Figure 8-6, XLSX file.

### The roles of *I*_MI_ and *I*_MI-T_ in the activity patterns of the LP neuron at different cycle periods

The bursting activity of follower pyloric neurons and, in particular, the LP neuron is exquisitely sensitive to the action of neuromodulation (Marder and Bucher, 2007; Stein, 2009; Harris-Warrick and Johnson, 2010), including proctolin (Nusbaum and Beenhakker, 2002; Daur et al., 2016). It is therefore natural to ask how the two currents *I*_MI_ and *I*_MI-T_ may differentially influence the LP bursting activity. Because we currently do not have an experimental method of separating the two components of *I*_Proc_ during the ongoing activity (no pharmacological blockers of these currents exist), we addressed this question in a computational model of the LP neuron (see Materials and Methods) based on the model developed by Taylor et al. (2009). Because the LP neuron bursts in response to periodic inhibition it receives from the pyloric pacemaker neurons, we drove the model with a periodic inhibitory synaptic input. We then examined the effect of the proctolin modulation on the bursting activity of the model neuron. *I*_MI_ and *I*_MI-T_ were adjusted to account for the spatial distance to the putative receptor location distal from the soma (see Materials and Methods). Note that the lack of slope dependence with small amplitude stimulations (Fig. 7) could be because of incomplete space clamp. All data shown from these modeling results are at steady state once the transient response of the model neuron has finished.

**Figure 9.**
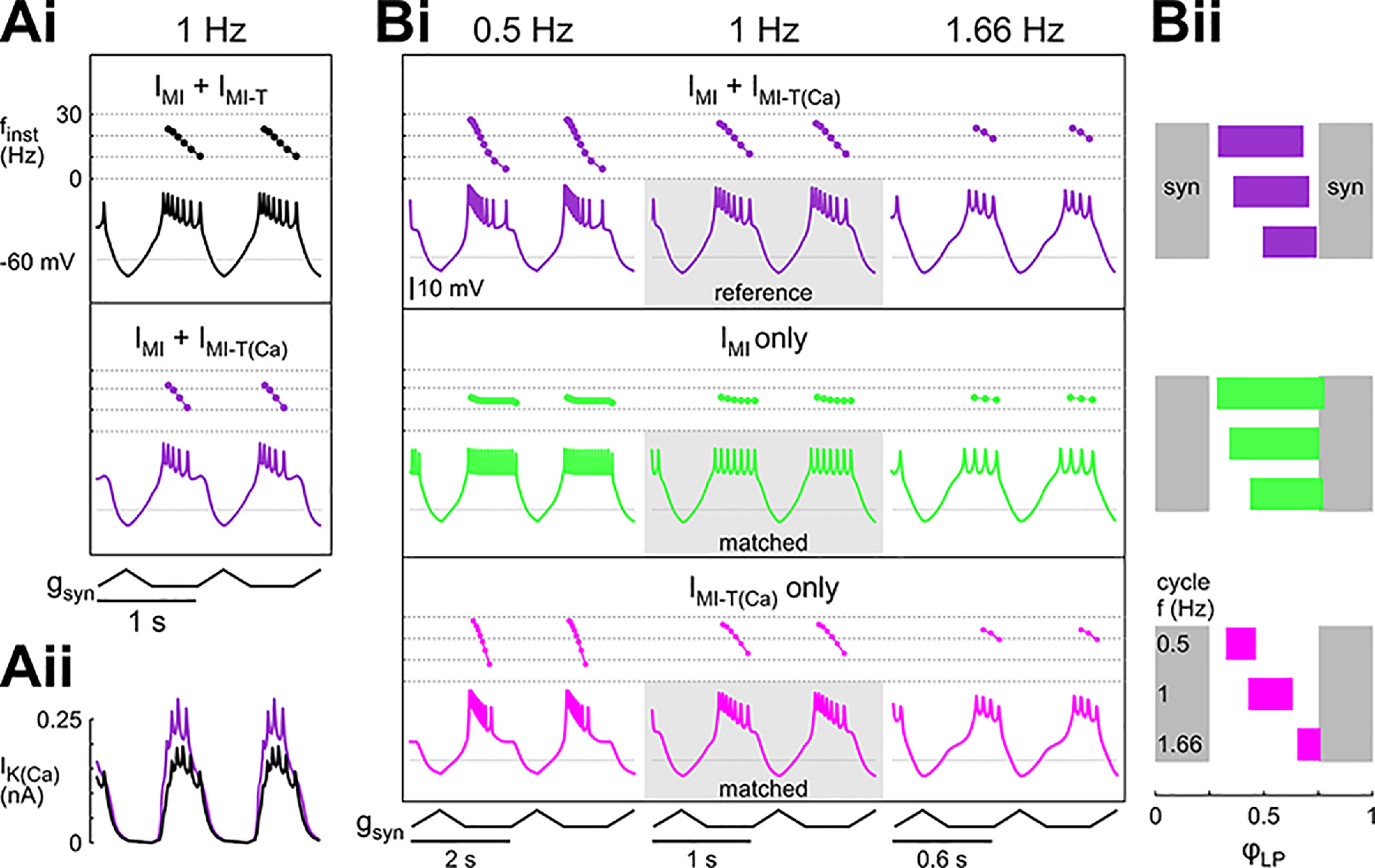
The frequency dependence of *I*_MI-T_ shifts the burst phases in a model of the LP neuron. LP model with *I*_K_ and *I*_Na_ in the axon compartment, leak current in both axon and soma/neurite compartment, and *I*_A_, *I*_h_, *I*_Ca_, *I*_K(Ca)_, *I*_MI_, and *I*_MI-T_ in the soma/neurite compartment. Calcium permeability is indicated by the subscripted addition of (Ca) to *I*_MI-T_. ***Ai***, Model voltage waveforms and the corresponding instantaneous spike frequencies (*f*_inst_) within a burst with *I*_MI_ and *I*_MI-T_ where *I*_MI-T_ is either contributing to the intracellular Ca^2+^ concentration (purple; *I*_MI-T(Ca)_) or not (black). The model received periodic inhibition (*g*_syn_) at 1 Hz. ***Aii***, Levels of *I*_K(Ca)_ when *I*_MI-T_ is either contributing to the intracellular Ca^2+^ concentration (purple) or not (black). The larger *I*_K(Ca)_ contributes to the earlier burst termination. The model equations and parameters are as described in Materials and Methods. ***B***, Contribution of the different components to the activity phases of the model neuron at different cycle frequencies. Currents were matched so that the model produced the same number of spikes per burst at 1 Hz (gray rectangles in ***Bi***). ***Bi***, Voltage trajectories and instantaneous frequencies within a burst at 0.5, 1, and 1.66 Hz when the model contained *I*_MI_ and *I*_MI-T(Ca)_ (purple), when the model contained only *I*_MI_ but not *I*_MI-T(Ca)_ (green), and when the model contained only *I*_MI-T(Ca)_ but not *I*_MI_ (pink). ***Bii***, Phase plots of the activity at different cycle frequencies. *I*_MI-T(Ca)_ had a substantially greater effect on phase than *I*_MI_ (Extended Data Fig. 9-1). Gray bars indicate the duration of the inhibitory synaptic current. In this panel, the model parameters for *I*_MI_ and *I*_MI-T_ were adjusted as follows: *I*_MI_ + *I*_MI-T(Ca)_: ${\bar{g}}_{MI}=0.227$, ${\bar{g}}_{MI-T}=2.27$; *I*_MI_ only: ${\bar{g}}_{MI}=0.995$, ${\bar{g}}_{MI-T}=0$; *I*_MI-T(Ca)_ only: ${\bar{g}}_{MI}=0$, ${\bar{g}}_{MI-T}=3.63$.

To see how Ca^2+^ permeability would influence the activity of the model neuron, we drove the LP neuron with synaptic input at a cycle frequency of 1 Hz, a typical average pyloric cycle frequency, and compared the effect of *I*_Proc_ = *I*_MI_ + *I*_MI-T_ at steady state, when the current was assumed to be permeable to Ca^2+^ (*I*_MI-T(Ca)_) and when it was not (Fig. 9*Ai*, black traces: not Ca^2+^ permeable, purple traces: Ca^2+^ permeable). We found that permeability to Ca^2+^ did not change the burst onset of the model neuron but shortened the burst duration so that the burst terminated before the arrival of the periodic inhibition. Consistent with the shorter burst, the number of spikes and the intra-burst spike frequency were both reduced, but there was no change in the burst structure in that the instantaneous spike frequency (*f*_inst_ = 1/interspike interval) decayed during each burst in both cases (Fig. 9*Ai*). To understand these effects, we compared the ionic currents of the models and found that the changes in the burst duration and spike number were because of the additional activation of the outward Ca^2+^-dependent K^+^ current, *I*_K(Ca)_, when the model *I*_Proc_ was assumed to be permeable to Ca^2+^ (Fig. 9*Aii*, *I*_MI-T(Ca)_, purple and pink traces), whereas the other ionic currents (which were not influenced by Ca^2+^ entry) were not affected (data not shown).

To examine the frequency-dependent effects of the two proctolin-activated currents, *I*_MI_ and *I*_MI-T_, we started with the model in Figure 9*A* (with Ca^2+^ permeability) at the cycle frequency of 1 Hz and tuned the levels of the modulatory currents to have seven spikes per burst. We refer to this as the reference model. We then changed the frequency of the periodic inhibitory synaptic input without changing the duty cycle, shape, or amplitude of this synapse. For simplicity, we only show the activity of the model neuron at two other frequencies, one lower (0.5 Hz) and one higher (1.66 Hz). Decreasing the cycle frequency slightly advanced the onset and end phases of the burst, and increased the number of spikes per burst, but did not affect the burst structure, in that *f*_inst_ followed the same decreasing trend within each burst (Fig. 9*Bi*, purple traces). Consistent with this, increasing the cycle frequency did the opposite. We then examined how proctolin modulation would influence LP activity if proctolin were to activate only *I*_MI_ or only *I*_MI-T_. However, simply removing *I*_MI-T_ from our reference model resulted in a complete absence of spiking at all three frequencies. Therefore, to do this comparison in the *I*_MI_ only case, we first increased the maximal conductance of *I*_MI_ so that, for the 1 Hz case, the number of spikes per burst matched the number of spikes of the reference model. Similarly, in the *I*_MI-T_ only case, we increased the maximal conductance of *I*_MI-T_ to achieve the same. These cases are denoted as “matched.” We then changed the frequency of the periodic input in each case and compared the effects with the reference case.

In the *I*_MI_ only case, changing the cycle frequency simply increased or decreased the number of spikes in the burst but the spike structure (*f*_inst_) of the model neuron remained relatively constant for the duration of the burst. In contrast, in the *I*_MI-T_ only case, both increasing and decreasing the cycle frequency shortened the duty cycle, but at 1.66 Hz, the burst was delayed in its onset, whereas at 0.5 Hz, it was advanced. In this case, the burst structure was also influenced by the change in cycle frequency. When cycle frequency was decreased, *f*_inst_ dropped dramatically within each burst (pink trace at 0.5 Hz), whereas it became somewhat more constant as cycle frequency was increased (pink trace at 1.66 Hz). The changes in the burst onset and end were reflected in the phase diagram of the model neuron (Fig. 9*Bii*). In the reference model (purple), there was a shift in the burst onset and a smaller shift in the burst end with increased cycle frequency. These shifts were reduced in the *I*_MI_ only case (green) and the phases were more similar across cycle frequencies. In contrast, the shifts became more pronounced in the *I*_MI-T_ only case (pink) and both the beginning and the end of the burst greatly shifted with increasing cycle frequency. Together, these modeling results indicate the ability of the two modulatory-activated currents to provide different influences on the burst structure, with *I*_MI_ producing a consistent burst duration and strength across all cycle frequencies and *I*_MI-T_ producing a dynamic change in burst structure across different cycle frequencies.

## Discussion

Oscillatory neuronal network activity can be stereotyped when conditions are stable (Bucher et al., 2005; Vajda et al., 2008) and can be highly dynamic when conditions are more variable, such as during development (Ben-Ari et al., 1989; Meister et al., 1991; Sharples and Whelan, 2017) or in injury states (Luther et al., 2003; Hamood and Marder, 2015). The activity transitions often depend on changes in neuromodulatory input that the networks receive. Such neuromodulatory inputs can generate activity patterns that depend on the original activity or excitation state of the network (Nusbaum and Marder, 1989; Doi and Ramirez, 2010; Sharples and Whelan, 2017), which in turn may depend on a neuron’s ionic conductances and synaptic inputs, as well as the specific ionic conductance(s) the neuromodulator regulates (Marder et al., 2014). At the individual neuron level, in neurons expressing oscillatory activity, neuromodulators may have variable effects because they may act differently depending on the neuron’s oscillation frequency.

Frequency-dependent interactions in the nervous system are typically thought of as changes in neural activity that depend on the input frequency, as is the case with short-term synaptic plasticity. Neuromodulators can modify the frequency dependence of the effects of such inputs (Ito and Schuman, 2008). However, the oscillatory membrane potential trajectory of a neuron can also affect how neuromodulators influence its activity in a frequency-dependent manner. When a neuron’s voltage is oscillatory, independent of whether the oscillation is imposed or intrinsic, the oscillation pattern affects how ionic currents activate, how they interact with each other, and consequently how they shape the voltage output of that cell and ultimately the activity of a network. For example, slow inactivation of an ionic current interacts with the rates of voltage change. Consequently, the mean level of an inactivating current could depend on the oscillation frequency because of incomplete recovery from inactivation. Neuromodulator-mediated changes of the gating properties of such transient currents will therefore have frequency-dependent effects. Currents activated by neuromodulators influence the voltage trajectory of the neuron, which alters the activation of other ionic currents as well as synaptic release. Thus, frequency dependence of neuromodulator effects on circuit activity may depend on activation of currents that are not directly targeted by the modulator.

Crustacean pyloric neurons are modulated by a host of endogenous peptides (Marder and Bucher, 2007). Most of these peptides increase the excitability of their target neurons, an effect that has been attributed to the fact that the peptides (as well as muscarinic agonists) convergently activate the fast persistent voltage-gated inward current *I*_MI_ (Golowasch and Marder, 1992; Swensen and Marder, 2000). Here, we show that one of these peptides, proctolin, additionally activates a transient current, *I*_MI-T_, in the follower LP neuron. *I*_MI_ and *I*_MI-T_ have similar voltage dependencies of activation but have different kinetics. Together, these two currents reproduce the LP neuron’s steady-state proctolin-mediated response to stimulation with different voltage waveforms, including voltage ramps and realistic waveforms at different cycle frequencies, in a computational model (Fig. 6). However, *I*_MI_ and *I*_MI-T_ make different contributions to the activity of the LP neuron. These distinct contributions arise from the effects of the voltage dynamics of the neuron on the activation and inactivation of the transient current, and interactions of both currents with other currents expressed by the LP neuron. One important interaction that we propose is with a Ca^2+^-activated K^+^ current (*I*_K(Ca)_). Large levels of *I*_K(Ca)_, among other potassium currents, have been shown to prevent neurons from oscillating, even when pacemaker currents were enhanced (Golowasch et al., 2017), and play a key role in regulating neuronal excitability (Haedo and Golowasch, 2006). Although our results do not provide proof of Ca^2+^ permeability of *I*_MI-T_, they provide strong evidence that this current is at least partly carried by Ca^2+^ ions (see below). Such Ca^2+^ permeability of *I*_MI-T_ would lead to the activation of *I*_K(Ca)_ (see Fig. 9*Aii*), with the important consequence that *I*_MI-T_, through its activation of *I*_K(Ca)_, will terminate the burst of spikes earlier than when only the persistent *I*_MI_ is active (Fig. 9*Ai*,*Bi*, green trace). Thus, it seems that the *I*_MI-T_ effect on the follower LP neuron’s bursting activity is 2-fold: it boosts early excitability by inducing postinhibitory rebound (see below), and it provides a brake on prolonged excitability because of its transient nature and the recruitment of an outward current.

### Persistent versus transient modulator activated currents

Persistent currents are thought to amplify excitatory synaptic inputs and cause membrane bistability (Jahnsen and Llinás, 1984; Lee and Heckman, 1998; Manuel et al., 2014). Like other persistent inward currents (Cantrell and Catterall, 2001; Tryba et al., 2006; Dunmyre et al., 2011), *I*_MI_ is known to act as a pacemaker current in the pacemaker neurons of the pyloric network (Bose et al., 2014). This pacemaker activity depends on a balance between *I*_MI_ and *I*_K(Ca)_ (Golowasch et al., 2017). However, follower neurons of this network, such as the LP neuron, express outward currents at such large amplitudes that they preclude *I*_MI_ from driving oscillatory activity (Golowasch et al., 2017). Although in the LP neuron the transient inward current *I*_MI-T_ has an even higher maximal conductance value than *I*_MI_, it does not elicit oscillatory activity in this neuron. This is probably because, in the absence of inhibitory input, the resting membrane potential of the LP neuron is fairly depolarized (Martinez et al., 2019), leading to the inactivation of *I*_MI-T_.

Consistent with the transient nature of *I*_MI-T_, the total proctolin-elicited current (*I*_MI_ + *I*_MI-T_) is more strongly activated by faster and positive-slope voltage ramps (Figs. 2-4). To get a better idea of how these currents are activated during natural ongoing oscillations, we measured them by voltage-clamping the LP neuron with a prerecorded realistic voltage waveform. To our surprise, the current measured with realistic waveforms had a negligible change in amplitude with faster voltage waveforms and became only slightly larger at 2 Hz compared with 0.5 Hz (Fig. 5*Bi*). Furthermore, the same voltage waveform applied at a higher frequency of 4 Hz elicited a much smaller current. This discrepancy could be because of the different activation and inactivation rates of *I*_MI-T_ in the voltage range used for ramps (−80 to +20 mV) compared with those of the realistic waveforms (−60 to −20 mV; Fig. 6). At the peak voltage of the realistic waveforms, the activation time constant for *I*_MI-T_ is relatively long. Thus, at fast cycle frequencies, *I*_MI-T_ cannot reach the same levels as at slower cycle frequencies and therefore the total proctolin current was smallest at the fastest cycle frequency we tested. Generally, slower time constants within a certain voltage range have been observed since the first description of the voltage-dependent transition rate constants by Hodgkin and Huxley (Hodgkin and Huxley, 1952; Ermentrout and Terman, 2010).

Is it possible that such a transient current, weakly activated in the normal voltage range of these cells, can significantly impact neuronal activity? To address the potential role of the modulator-activated currents on the activity of the LP neuron we resorted to computational modeling. During its normal biological activity, the LP neuron receives strong inhibitory input from the pyloric pacemaker neurons and rebounds from this inhibition to produce a burst of action potentials. Because it is fast and non-inactivating, *I*_MI_ influences the spiking frequency of the LP neuron independent of the frequency of the synaptic inhibition. In contrast, the effect of *I*_MI-T_ is different depending on the frequency of the synaptic input. At low frequencies (e.g., 0.5 Hz) the current is strongly activated and generates a postinhibitory rebound but inactivates within the duration of a burst. Consequently, the burst terminates before the onset of the next cycle of inhibition (Fig. 9 *Bi*, pink and purple traces). At high frequencies (>1 Hz), however, the rapid cycling of the voltage prevents full recovery from inactivation and the total available *I*_MI-T_ is lower. This leads to a delayed onset of action potential firing at high frequencies. Together, these two neuromodulator-activated currents lead to shorter bursts with smaller duty cycles and higher transient spiking frequencies at all synaptic input frequencies compared with the case if only the persistent current were activated. As for how the two currents influence the activity phases of the LP neuron, the persistent *I*_MI_ supports consistent spiking when LP is not inhibited, whereas the transient *I*_MI-T_ allows for a modulation of the phases of burst onset and termination at different input frequencies (Fig. 9*B*). The activity phase of the LP neuron is known to regulate the pyloric cycle period through its synaptic inhibition of the pyloric pacemaker group (Johnson et al., 2011). Additionally, the same study showed that monoamines differentially modulate the LP activity phase, which influences the impact of the synaptic feedback from the follower LP neuron on the cycle period. However, the mechanism by which amines regulate the activity phase most likely does not involve *I*_MI-T_ since those amines do not activate *I*_MI_. On the other hand, we predict that other neuromodulators, which activate *I*_MI_ in LP and other neurons, activate *I*_MI-T_ as well.

Most of the previous studies on peptide-activated currents in the STG measured the response as a difference current at steady state using voltage-step protocols or slow ramps (Li et al., 2018). Because the peptide-activated currents are relatively small compared with the large outward currents in STG neurons (often in hundreds of nA), it is extremely difficult to measure transient difference currents even if outward currents are pharmacologically blocked (which is never perfect), as even small variations in the measurement done at different times (control vs modulator saline) can lead to significant errors. We found that ramp protocols partially remedy this problem (by partially inactivating the large outward currents) and provide consistent modulator-elicited difference currents measurements over many repetitions. Since we used relatively fast ramps, we were able to measure transient currents before complete inactivation. Because of the time-dependent inactivation of *I*_MI-T_ during the positive ramp, the total current activated by proctolin was larger at faster depolarization rates and the peak of this current shifted to more depolarized membrane potentials: depolarized voltages were reached in a shorter time. It is noteworthy, however, that transient currents are often not easily identifiable with ramp protocols (but see Park et al., 2013). Nevertheless, one advantage of using ramp (over step) protocols is that a range of voltages can be sampled continuously. Additionally, changing the slope of a voltage ramp mimics the voltage changes that an oscillating neuron experiences during each cycle of realistic oscillatory activity, which can help gain insight into the physiological relevance of the current. On the other hand, an important drawback of using ramps is that an incomplete inactivation during a positive voltage ramp can influence the current measured on the negative voltage ramp (Fig. 2). A clear indication of this drawback was that parameter differences during negative ramps were no longer present in the steady state measurements during ramp-and-hold protocols (Fig. 4).

Rodriguez et al. (2013) identified two voltage-dependent transient currents activated by a neuropeptide in an STG gastric mill circuit neuron. These currents were not evident with ramp protocols because of inactivation and could only be observed with voltage step protocols. Rodriguez et al. (2013) indicated the presence of the faster of the two currents but did not further characterize it. The slower current, *I*_Trans-LTS_, shares some similarities with *I*_MI-T_ in that both are transient, modulator-activated, voltage-gated currents that are probably carried by a combination of Na^+^ and Ca^2+^ ions. Furthermore, both inactivate completely during slow voltage ramps. However, *I*_Trans-LTS_ is evident in voltage-step-elicited raw current recordings because it activates slowly (500 ms), whereas *I*_MI-T_ is much faster to activate and also inactivates rapidly. It is therefore unlikely that *I*_MI-T_ is the same current as *I*_Trans-LTS_, but it is possible that *I*_MI-T_ is the same as the fast transient current observed, but not further characterized, by Rodriguez et al. (2013).

*I*_Trans-LTS_, enables postinhibitory rebound bursting. Such rebound bursting has been shown to be important in the activity of a number of neurons (Llinas, 1988; Getting, 1989), including the rebound properties sometimes required to generate network oscillatory activity (e.g., half-center oscillators; Rodriguez et al., 2013), bistability (Hounsgaard et al., 1988), and even as a form of intrinsic short-term memory mechanism (Goaillard et al., 2010). As mentioned above, our modeling results show that *I*_MI_ contributes to continuous spiking in the LP neuron when it is not inhibited, whereas *I*_MI-T_ can produce a postinhibitory rebound significantly larger than the baseline activity, which would contribute to this neuron’s role in the pyloric CPG, as well as to activating its target muscles. These two modes of bursting (periodic inhibitory pauses of baseline activity vs significant rebound from inhibition) can have vastly different effects on postsynaptic neurons. For example, a recent report shows that dopaminergic neurons in the substantia nigra generate robust postinhibitory rebound bursts following inhibition by striatal neurons, whose transmissions target both GABA-A and GABA-B receptors. In contrast, inhibition of the same neurons by globus pallidus neurons, which activate only GABA-A receptors, does not generate rebound activity, but just pauses ongoing spiking (Evans et al., 2020). Importantly, only the former postinhibitory rebound bursts produce phasic release of dopamine in the striatum by the nigral neurons, which is a key factor in striatal synaptic plasticity and reinforcement learning (Yagishita et al., 2014; Shindou et al., 2019).

### Voltage dependence and the role of calcium

Previous studies that examined peptide modulation of STG neurons were mostly done in the presence of Ca^2+^ blockers (Golowasch and Marder, 1992; but see Rodriguez et al., 2013), which may explain why they did not find evidence of *I*_MI-T_. Although the slow inactivation of *I*_MI-T_ over multiple cycles (Fig. 3) could in principle be accounted for by an additional slow inactivation gate, such a slow gating property would predict distinct time constants of *I*_MI-T_ reduction at different depolarization frequencies. However, Figure 3*A*,*C* shows that no such difference can be detected. A simple Goldman–Hodgkin–Katz model of *I*_MI-T_ as a Ca^2+^ current indicated that intracellular accumulation of Ca^2+^ is sufficient to explain the slow inactivation of the current over many seconds (Fig. 6*C*). This is consistent with the previously reported Ca^2+^ dependence of *I*_MI_. *I*_MI_ appears to be sensitive to both extracellular and intracellular Ca^2+^, and the intracellular Ca^2+^ is likely modified by Ca^2+^ flux through the *I*_MI_ channels themselves (Gray and Golowasch, 2016; Gray et al., 2017). Our findings suggest that *I*_MI-T_ may be similarly permeable to Ca^2+^ and perhaps even depend on it for its activation and inactivation. Altogether, several neuromodulator-activated and voltage dependent currents appear to be present in several STG neurons. They all bear resemblance to the better characterized non-inactivating *I*_MI_ (Golowasch and Marder, 1992; Swensen and Marder, 2000, 2001; Gray and Golowasch, 2016; Gray et al., 2017), but some express inactivation (*I*_MI-T_, *I*_Trans-LTS_), and all seem to vary in activation and inactivation kinetics. It is not known, but remains possible, that all convergently respond to multiple neuromodulators like *I*_MI_ does (Swensen and Marder, 2000, 2001). This would suggest that the ion channels that produce *I*_MI-T_ and *I*_MI_, and perhaps also *I*_Trans-LTS_, could all be isoforms of the same channel. It is well known that ion channel isoforms can be generated by various mechanisms, including alternative splicing (Soldatov, 1994) and gene duplication (Piontkivska and Hughes, 2003), and that isoforms may be unevenly distributed within tissues (Tellez et al., 2006). The isoform composition of an ion channel gives it distinct isoform-dependent characteristics, e.g., electrophysiological properties and blocker sensitivity (for review, see Hameed, 2019), or pH sensitivity (Khan et al., 2006). Similarly, channel isoforms have already been identified in the transcriptomes of *C. borealis* and the lobster *Homarus americanus* for multiple ion channel types (Northcutt et al., 2016). This is all consistent with the possibility that the various voltage-gated ionic current modulated by neuropeptides in this system are isoforms within an ion channel family, whose identity and precise distribution has yet to be determined.

Overall, the transient nature and resulting frequency dependence, and Ca^2+^ permeability of *I*_MI-T_ give this current the potential to widely influence the activity of a neuron. Since LP is the only follower neuron that synapses onto the pacemaker group, frequency dependent changes in LP activity could in turn feed back to the pacemakers and stabilize the pyloric rhythm at a preferred frequency. It remains to be shown if *I*_MI-T_ can be activated by other neuropeptides, similar to *I*_MI_, and if and to what extent *I*_MI-T_ is present in other neurons.

10.1523/ENEURO.0338-21.2021.f9-1 Extended Data Figure 9-1 Data for panel ***Bii***. Download Figure 9-1, XLSX file.

## Synthesis

Reviewing Editor: Alexey Semyanov, Shemyakin-Ovchinnikov Institute of Bioorganic Chemistry Decisions are customarily a result of the Reviewing Editor and the peer reviewers coming together and discussing their recommendations until a consensus is reached. When revisions are invited, a fact-based synthesis statement explaining their decision and outlining what is needed to prepare a revision will be listed below. The following reviewer(s) agreed to reveal their identity: Bruce Johnson.

Two reviewers have seen the manuscript, and they both liked it. Nevertheless, they made several comments which you may be able to address.

Reviewer #1

This is a very well-written manuscript that thoroughly describes the contributions of two modulator-activated currents to regulating activity of an oscillatory neuron. Importantly, the authors provide a mechanism for state-dependent modulatory actions on oscillatory neurons, namely the frequency-dependence of an inactivating current.

Major comments.

Line 388-394/figure 7 results. I’m having difficulty reconciling the lack of slope dependence across ramps from -60 to -20 mV (realistic voltage range) with the results presented in figure 9. In figure 9, the actions of IMI-T vary across frequency, which I thought indicates a slope dependence of the current amplitude. Are the small (n.s.) differences in Imax across slopes (figure 7) sufficient to explain the frequency dependence in figure 9? I think this needs to be explicitly addressed. How do the data in figure 7 and 9 fit together?

Minor Comments.

Line 2 (also 18). Can the authors be more explicit that the “frequency-dependent effects” or “frequency-dependent actions” of neuromodulators refers to their dependence on the oscillatory frequency of the neurons? Line 6 sort of addresses this as “temporal properties of neuronal activity” but I think all of this is too implicit. I think frequency-dependent effects of neuromodulators is likely to make many readers think that the authors are referring to potential frequency-dependence of release of neuromodulators, or synaptic frequency-dependence. This is such a big point of the study, I think it is worth being more explicit. Maybe referring to it as bursting or oscillation frequency throughout the abstract would help? Something similar to line 56-57 would be very helpful in the abstract.

Line 9 change know to known.

Line 11. Insert “increasing” before ramp slope.

Line 405 (and 900). Are the traces in Fig. 8E in Cd++? Would be helpful to explicitly indicate why there was no inactivation here.

Line 426 and 433. It would be useful to indicate the color code for Ca++ permeable or not in the text.

Line 479. insert “a” between “on neuron’s"

Line 539. Please provide context for the different activation/inactivation rates used for -60 to -20 mV. Are there examples in the literature of slower rates across a voltage range with faster rates above and below that range?

Line 613. Delete “do"

Line 626. The discussion feels like it ends abruptly. Maybe a brief summary/conclusion?

Line 788. Change “on lvn” to “in the lvn recording"

Line 792. Comma after 40 mV

Line 793. Change “ranging from -60 mV to -20 mV” to “from a trough potential of -60 mV to a peak potential of -20 mV”. As currently written it sounds to me like the potential varies across experiments.

Line 805. Change “of” to “from"

Line 878. The end of the title for this figure legend is missing.

Line 914. A more informative title for this figure would be useful. It is not simply a model of the LP neuron, it is presenting an important result about the contributions of the two currents (in a model of the LP neuron).

Figures

I recommend increasing the size of all subscripts in figures.

Fig. 6C. The same thickness of the red and black lines makes it difficult to see the results. It might be easier to see if the line in the background was thicker than the line in the foreground. This is less of a problem for 6Ai and Bii as the traces are not as compressed as in 6C, but it would be easier to see both traces in all three sets if a thick black trace was behind a thin red trace for example.

Fig. 9. It would be helpful to the reader to have labels or legends for calcium permeable/not included in the figure.

Reviewer #2

General Comments:

This is a very interesting paper that carefully dissects a previously noted modulatory current

activated by proctolin in an identified neuron in the crab stomatogastric system. It addresses the ionic current mechanisms by which neuromodulators can determine the intrinsic properties of neurons active in neural networks such as central pattern generators. The paper thoughtfully combines newly gathered experimental data to build a computational model of the modulatory action at the ion channel level, and then tests predictions of the model with further experimental data. The paper is a good example of the best interactive use of experimental and computational approaches to answer mechanisms of neuromodulatory shaping of neural network activity. This is a dense paper with the modelling and electrophysiological data, but it is well written and mostly guides the reader though the details well. I only have moderate and minor comments on the manuscript.

Moderate comments:

Methods -Line 129: It may be a safe assumption that “ current measured in the negative ramp excluded currents that inactivated during the positive ramp", but could other currents not related to the proctolin effect like Ih be deactivating slowly enough to still be a factor during the negative ramp? Line Results 259: - Sometimes the text descriptions of the figure parts go by too quickly to understand all the figure parts shown. I suggest making sure that all figure parts are briefly described or at least referred to in the Results text so the reader can rely less on the figure legends to understand detailed figures. For example, mention Fig 2A, B specifically in line 263 instead of just Fig 2.

Discussion: Line 513- The Ca activation of IKCa as break on excitability needs more context from the literature showing that this happens in oscillating networks. Maybe move up the reference to Golowasch et al 2017 here for the context.

Line 554- Amines can also shape the LP activity phases though action on ionic currents to have an impact on network activity. Consider mentioning your results relate to other neuromodulators too. See Johnson et al 2011 J Neurophysiol 105:293.

Line 624: The isoform idea is interesting but only brought up in a sentence at the end of the paper. It’s important enough to put in the abstract and deserves a little more discussion here. Are there other examples in the literature to provide support for this idea?

Minor comments;

Line 9- should be “known"

Line 613 delete “do”.
